# Emergence of Frank–Kasper Phases from Chemically Simple Block Copolymer: Poly(ethylene oxide)-*block*-polyisoprene and Its Dry-Brush Blends

**DOI:** 10.1021/acs.macromol.5c03133

**Published:** 2026-02-02

**Authors:** Zi-En Huang, Yung-Chuan Chuang, Yung-Chen Lin, Yu-Chuan Sung, Kai-Wei Luo, Jing-Cherng Tsai, Hsin-Lung Chen

**Affiliations:** † Department of Chemical Engineering, 34881 National Tsing Hua University, Hsinchu 300044, Taiwan; ‡ Department of Chemical Engineering, 34915 National Chung Cheng University, Chiayi 621301, Taiwan

## Abstract

The Frank–Kasper (FK) phases, also known as tetrahedrally close-packed structures, represent a unique class of ordered morphologies characterized by large unit cells with multiple nonequivalent lattice sites. To date, only a limited number of linear AB-type block copolymers (BCPs) have been shown to form FK phases. Here, we systematically investigate a sphere-forming poly­(ethylene oxide)-*block*-poly­(1,4-isoprene) (PEO-*b*-PI) with a conformational asymmetry parameter ε ≈ 1.26 and identify it as a new linear diblock system capable of forming the FK σ phase. In the neat PEO-*b*-PI, an abrupt enlargement of micelle size was observed across the BCC-to-σ lattice transition, despite a reduction in diblock molecular weight, indicating the pronounced influence of lattice symmetry on micelle dimensions at the onset of FK phase formation. To further expand the accessible FK phase regime, we employed a dry-brush blending strategy by incorporating homopolymer PEO (h-PEO) into a BCC-forming PEO-*b-*PI. With increasing h-PEO content, the blends exhibited a lyotropic BCC → σ → C14 → C15 phase transition sequence consistent with the theoretical predictions. Detailed structural analysis revealed systematic variations in micelle characteristics with h-PEO composition, arising from the interplay among different free energy components. Notably, the FK phases in this system were stabilized over an unusually broad composition window, allowing the Laves C15 phase to persist up to nearly symmetric compositions, significantly beyond the stability limits reported for conventional BCP/homopolymer blends.

## Graphical abstract

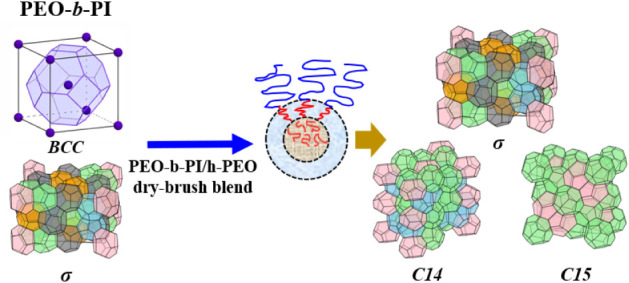

## 1 Introduction

Block copolymers (BCPs) of highly asymmetric composition can self-assemble into spherical micelles in the melt, with the shorter A block forming the core and the longer B block constituting the corona. The size of an isolated spherical micelle reflects a balance between the interfacial free energy penalizing A–B contact at the core–corona interface and the increase of conformational free energy arising from chain stretching to reduce the interfacial area per chain at the junction.^1^ In concentrated melts, the requirement of uniform segmental density imposes a space-filling constraint. Because spheres cannot tile the space without voids, the micelles and even their cores may deform toward polyhedral shapes that approximate the Voronoi cells of the lattice.^1–5^ The thermodynamically stable packing lattice is thus determined by the configuration that minimizes the total free energy penalty arising from these geometric distortions, reflecting the delicate balance among interfacial tension, chain deformation, and the intermicellar interaction relating to the coronal chain interdigitation at the intermicellar boundaries.^3,6^

BCP micelles in the melt state most commonly pack into a body-centered cubic (BCC) lattice, in which micelles deform into truncated octahedra to achieve space filling with minimal conformational free energy penalty associated with deviations from uniform chain dimensions.^1,3,4,6^ However, an increasing number of studies have demonstrated that spherical BCP micelles can also assemble into more complex packing lattices beyond BCC, most notably the Frank–Kasper (FK) phases.^7–10^ These geometrically intricate structures comprise multiple inequivalent polyhedral coordination environments with distinct coordination numbers, providing an efficient yet inherently frustrated mode of space filling.^11–13^ Unlike the uniform environment of the BCC lattice, FK phases accommodate variations in micelle size and local curvature through the coexistence of inequivalent sites, enabling a cooperative redistribution of interfacial, elastic, and geometric free energy contributions.^3,4,13^ The spontaneous emergence of FK phases in BCP systems therefore challenges the classical paradigm of uniform micelle packing and highlights how complex order can arise in single-component soft-matter assemblies.

Despite the broad diversity of FK phase identified in metallic alloys, only four, i.e., σ, A15, and the Laves C14 and C15 phases, have been experimentally observed in BCP systems to date.^7–10^ The conformational asymmetry, quantified by the parameter ε = *b*_*A*_/*b*_*B*_, where *b*_*A*_ and *b*_*B*_ are the statistical segment lengths of the core- and coronal-forming blocks (normalized to a reference volume), has been recognized as a critical molecular parameter governing the accessibility of FK phases in BCP melts.^13–15^ Systems with ε > 1 exhibit conformational asymmetry in which the majority B block is stiffer than the minority A block. Under an equivalent stretching, B block incurs a higher elastic free energy penalty than its softer A counterpart; as a result, the system preferentially bends the interface toward the A block to reduce the overall elastic cost, effectively stabilizing spherical micelles even at elevated volume fractions of the minority A component. Self-consistent field theory (SCFT) predicts that, whereas conformationally symmetric BCPs transform from spherical to cylindrical morphologies upon increasing core volume fraction, conformationally asymmetric BCPs can sustain spherical phase to higher core fractions, thereby accessing the regime where FK phases become thermodynamically favored.^13,15^

Experimentally, FK phases have been observed in BCPs where pronounced conformational asymmetry is introduced not only by disparities in chain rigidity but also by complex molecular architectures, such as miktoarm star.^16–18^ Beyond the chemical synthesis of BCPs with intrinsically high conformational asymmetry, recent studies have shown that FK phase formation can also be promoted through physical blending strategies.^19,20^ These include blending BCPs with homopolymers localized in either the core or corona,^20–24^ mixing with other BCP species,^19,25–27^ or selectively incorporating metal salts into the micelle core.^28,29^

To our knowledge, only a few linear diblock copolymers have been reported to exhibit the ability to form FK phases. Notable examples include polyisoprene-*block*-polylactide (PI-*b*-PLA, ε = 1.15)^7^ and poly­(ethyl ethylene)-*block*-polylactide (PEE-*b*-PLA, ε = 1.30),^14^ both of which form the σ phase, as well as poly­(dodecyl acrylate)-*block*-polylactide (PDDA-*b*-PLA, ε = 1.85)^9^ and oligo­(γ-dodecyl-α-hydroxyglutaric acid)-*block*-oligo­(lactic acid) (oDGA-*b*-oLA, ε = 1.88),^30^ which form the A15 phase. A15 and σ were previously regarded as the only thermodynamically stable FK phases accessible in linear diblock systems. However, this view was reconsidered following the work of Jeon et al., who demonstrated the formation of the Laves C14 phase in a poly­(dimethylsiloxane)-*block*-poly­(2,2,2-trifluoroethyl acrylate) (PDMS-*b*-PTFEA) system exhibiting high conformational asymmetry (ε = 2.2).^10,31^ In this case, application of the diblock foam model predicted that the C14 phase became thermodynamically stable at relatively low core volume fractions.

Despite this discovery, the thermodynamically stable Laves C14 and C15 phases have been primarily accessed through physical blending strategies.^26,32–34^ One plausible approach involves blending a BCP with the corresponding homopolymer of the core-forming block.^32,34^ When the homopolymer has a molecular weight comparable to that of the minority block (i.e., α = *M*_*h‑A*_/*M*_*b‑A*_ ≈ 1), it preferentially localizes near the center of the micelle core, a phenomenon known as “dry-brush mixing.” This localization effectively enlarges the core size without substantially decreasing the interfacial curvature, thereby preserving the spherical morphology at relatively high core volume fractions and facilitating the formation of stable FK phases, including the Laves phases.^35^

In this study, we report a new linear diblock system, poly­(ethylene oxide)-*block*-poly­(1,4-isoprene) (PEO-*b*-PI), capable of forming FK phase. Both PEO and PI are well-established and widely studied polymers, making this system particularly compelling as it demonstrates the formation of complex spherical phases using chemically conventional and synthetically accessible building blocks. The PEO-*b*-PI samples investigated span PEO core volume fractions (*f*_*PEO*_) from 0.124 to 0.258. We found that the FK σ phase emerged when *f*_*PEO*_ exceeded approximately 0.25, which is attributable to the system’s intrinsic conformational asymmetry, characterized by the value of ε = *b*_*PEO*_/*b*_*PI*_ ≈ 7.3 Å/5.8 Å = 1.26.^14,34^

To further extend the accessible complex spherical phase regime, we applied a dry-brush blending strategy by incorporating homopolymer PEO (h-PEO), with molecular weight matching that of the core-forming PEO block, into a BCC-forming PEO-*b*-PI. The resulting blends exhibited a lyotropic phase transition sequence of BCC → σ → C14 → C15 with increasing h-PEO content. This progression was consistent with the SCFT predictions^35^ and mirrored previous experimental observations in other blend systems.^32,34^ However, a notable distinction in the PEO-*b*-PI/h-PEO blends is the significantly broader stability window of complex spherical phases, particularly the Laves C15 phase, which persisted up to a total PEO volume fraction of 0.52 without macrophase separation. This enabled the formation of the FK phase near symmetric composition, an uncommon feature in the conventional BCP systems.

In addition to mapping the phase behavior, we identified two notable structural features. First, in the neat PEO-*b*-PI system, an abrupt enlargement of micelle size was observed across the BCC-to-σ lattice transition, demonstrating the pronounced influence of lattice symmetry on micelle dimensions, particularly at the onset of the FK phase. Second, in the dry-brush blends, the micelles exhibited subtle but systematic variations in structural characteristics with increasing h-PEO content, which arose from the delicate interplay among different free energy components. These findings provide new insight into how dry-brush blending can be exploited to stabilize complex spherical phases in chemically simple BCP systems.

## 2 Experimental Sections

### 2.1 Synthesis and Characterizations of PEO-*b*-PI Diblock Copolymer

The PEO-*b*-PI was synthesized through a two-step process. First, the PI block was prepared via living anionic polymerization of isoprene initiated by *tert*-butyllithium (*tert*-BuLi) in benzene at room temperature. The resulting PI block primarily consisted of 1,4-addition units with a minor fraction of 3,4-addition, as determined by ^1^H nuclear magnetic resonance (NMR) spectroscopy. Subsequently, an excess of the living PI chains was coupled with tosyl-terminated PEO. After quenching the reaction with methanol, the product was purified by column chromatography using a 1:1 ethyl acetate/hexane eluent to remove excess PI, yielding the desired PEO-*b*-PI sample.

All the BCP samples were characterized by gel permeation chromatography (GPC) and ^1^H NMR spectroscopy. The detailed synthetic procedures and molecular characterizations of the representative PEO-*b*-PI product are presented in the Supporting Information (SI). Table 1 lists the molecular characteristics of the PEO-*b*-PI samples synthesized.

**Table 1:** Molecular Characteristics of the PEO-*b*-PI Samples Studied

Sample^a^	*M*_n,PEO_ (g/mol)^b^	*M*_n,PI_ (g/mol)^c^	*N* ^d^	*Đ* ^e^	*f*_*PEO*_ ^f^	1,4-addition PI (mol %)^g^
PEO^1.3^-*b*-PI^3^	1,300	3,000	61	1.04	0.258	91.7
PEO^1.3^-*b*-PI^3.06^		3,060	62	1.07	0.255	92.0
PEO^1.3^-*b*-PI^3.15^		3,150	63	1.07	0.249	92.3
PEO^1.3^-*b*-PI^4.5^		4,500	82	1.04	0.188	92.2
PEO^1.3^-*b*-PI^5^		5,000	89	1.04	0.173	91.3
PEO^1.3^-*b*-PI^5.4^		5,400	95	1.04	0.162	92.1
PEO^1.3^-*b*-PI^6^		6,000	103	1.04	0.148	91.8
PEO^1.3^-*b*-PI^7.35^		7,350	122	1.03	0.124	91.9

a Each BCP sample is denoted as PEO^*n*^-*b*-PI^*m*^, where *n* and *m* represent the molecular weights in the unit of kg/mol of the PEO and PI blocks, respectively.

b Number-average molecular weight of PEO block.

c Number-average molecular weight of PI block.

d The degree of polymerization of the diblock relative to a reference volume of 118 Å^3^.

e Polydispersity index determined by GPC.

f Volume fraction of PEO block.

g Mole percentage of 1,4-addition units in the PI block determined by ^1^H NMR.

### 2.2 Preparation of PEO-*b*-PI/h-PEO Blends

The blends of PEO-*b*-PI and h-PEO were prepared by solvent casting. The components were dissolved in tetrahydrofuran (THF) at room temperature. The resulting solution was then dried under vacuum at 60 °C for 4 h to obtain the solvent-cast blends. To remove any residual solvent history, the samples were heated at a rate of approximately 7 °C/min to 140 °C, a temperature well above the order–disorder transition (ODT) temperature (*T*_*ODT*_). After heating, the samples were cooled to 30 °C and subsequently stored at this temperature for several days prior to small-angle X-ray scattering (SAXS) measurements. The blend compositions are reported as nominal values, expressed either as the overall volume fraction of PEO (*f*_*PEO*_) or as the volume fraction of h-PEO (*f*_*h*‑*PEO*_), as defined by the feed ratios used during sample preparation.

Except for PEO-*b*-PI/h-PEO blends with high h-PEO content, where macrophase separation occurred, all samples remained essentially amorphous after equilibration at 30 °C; therefore, the self-assembled structures were not influenced by the crystallization of the PEO components. This was verified by wide-angle X-ray scattering (WAXS) measurements performed on samples equilibrated at 30 °C (Figure S4 in the SI). Both the neat PEO-*b*-PI and the dry-brush blend with lower h-PEO content exhibited only a broad amorphous halo, indicating that the PEO components forming the micellar cores remained amorphous. The suppressed crystallizability originated from the strong spatial confinement of PEO within discrete microdomains, where crystallization required homogeneous nucleation. As shown previously, the temperature required to initiate homogeneous nucleation of PEO confined in spherical domains can be as low as −30 °C, well below the temperatures explored in this study.^36^ Consequently, the ordered structures observed by SAXS were not perturbed by PEO crystallization.

### 2.3 Small-Angle X-ray Scattering (SAXS) Measurement

Temperature-dependent SAXS measurements were conducted at TLS 23A1 SWAXS and TPS 25A1 coherent X-ray scattering beamlines of the National Synchrotron Radiation Research Center (NSRRC), Hsinchu, Taiwan. The energy of the employed monochromatic radiation was 15 keV, corresponding to an X-ray wavelength (λ) of 0.083 nm. The 2D scattering patterns were recorded with a PILATUS 1M detector at TLS 23A1 and an EIGER 16M detector at TPS 25A1. The instrument covered the modulus of the scattering vector *q* of approximately 0.07 to 4 nm^–1^, where *q* is defined as *q* = 4π/λ sin­(θ/2), with λ and θ being the X-ray wavelength and the scattering angle, respectively.

For the temperature-dependent SAXS measurements, samples were heated or cooled in 10 °C increments, with a 5 min isothermal hold at each temperature. The temperature ramp rate between set points was maintained at approximately 5 °C min^–1^, corresponding to an average effective heating/cooling rate of about 1.4 °C min^–1^ over the entire thermal cycle. All scattering intensity profiles were corrected for background contributions from air and the empty sample cell.

## 3 Results and Discussion

### 3.1 Phase Behavior of the PEO-*b*-PI Melts

A series of PEO-*b*-PI diblock copolymers, which formed the micelles with PEO as the core, were synthesized. Each BCP sample is denoted as PEO^*n*^-*b*-PI^*m*^, where *n* and *m* represent the molecular weights (in kg/mol) of the PEO and PI blocks, respectively. The molecular weight of the PEO block was fixed at 1300 g/mol, while the overall composition of the BCP was tuned by varying the molecular weight of the PI block. Figure 1(a) shows the temperature-dependent SAXS profiles of PEO^1.3^ *-b*-PI^4.5^ with *f*_*PEO*_ = 0.188, collected during a stepwise heating process. Between 30 and 50 °C, the SAXS pattern exhibited a series of reflections with the peak position ratio of 1:2^1/2^:3^1/2^:5^1/2^, consistent with micelles arranged in a BCC lattice. Upon further heating, the BCC phase underwent an ODT at approximately 60 °C.

**Figure 1:**
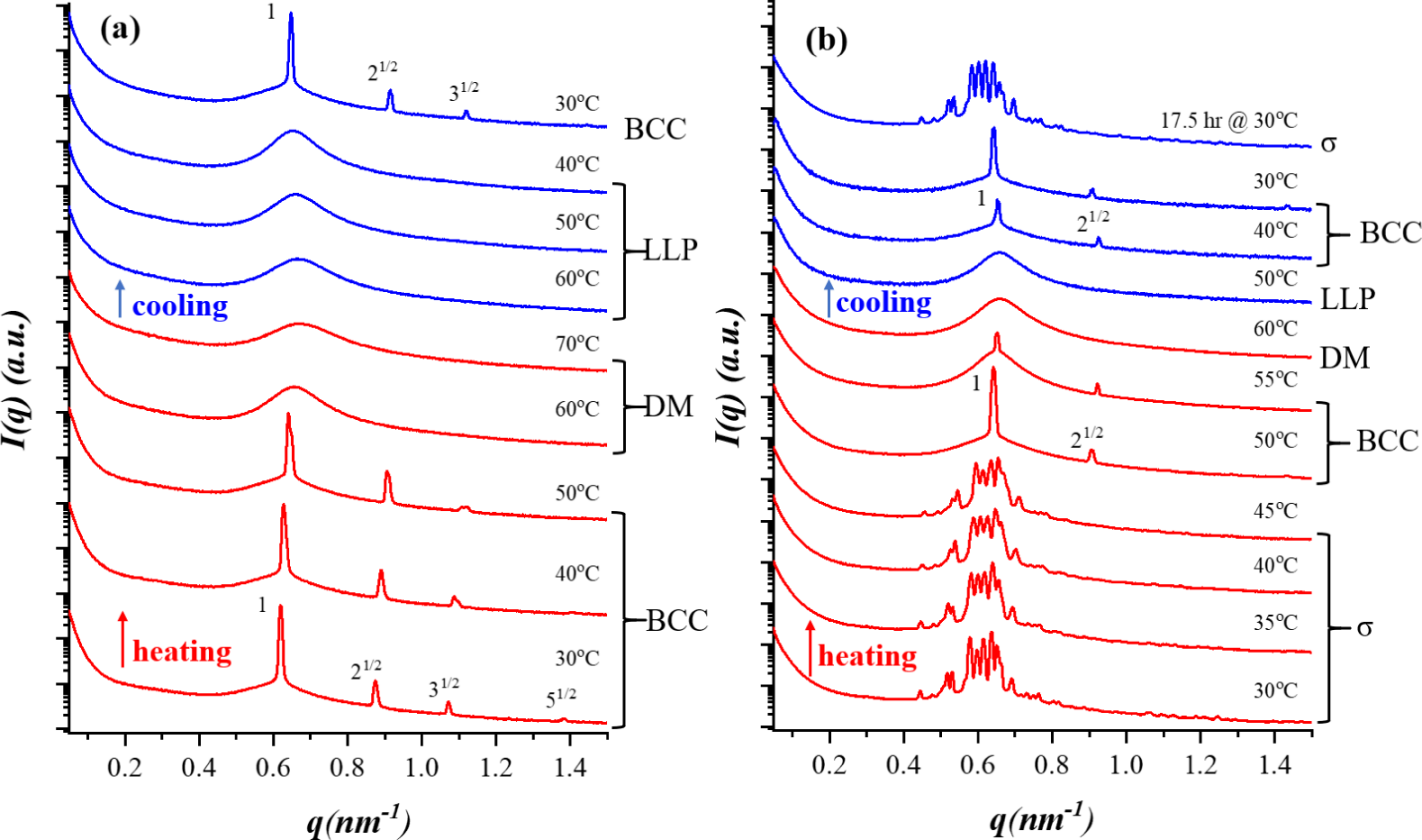
Temperature-dependent SAXS profiles of PEO-*b*-PI with (a) *f_PEO_* = 0.188 and (b) *f_PEO_* = 0.255. The red and blue curves correspond to the scattering profiles collected during the heating and subsequent cooling processes, respectively. The structural phases appearing within different temperature ranges are indicated in the figure, where DM and LLP denote the disordered micelle and liquid-like packing phases, respectively. The FK σ phase was observed in PEO^1.3^-*b*-PI^3.06^ with a higher PEO volume fraction (*f_PEO_* = 0.255).

In contrast, PEO^1.3^-*b*-PI^3.06^, which has a higher *f*_*PEO*_ of 0.255, showed a more complex SAXS pattern at 30 °C (Figure 1(b)), characterized by a large number of peaks. This scattering pattern is consistent with the FK σ phase, with reflections indexed to the *P*4_2_/*mnm* space group corresponding to a tetragonal unit cell with the lattice parameters *a* = 44.78 nm and *c* = 23.69 nm (*c*/*a* = 0.529). Upon heating to 50 °C, the σ phase transformed into the BCC phase. This thermotropic transition was attributed to the reduction of the χ parameter at elevated temperatures, which diminished the relative contribution of interfacial free energy. Consequently, the micelles favored BCC packing, which imposed a lower conformational free energy penalty. The observed σ-to-BCC transition with increasing temperature (or decreasing χ) aligned with the SCFT predictions.^13^

The system became disordered at 60 °C. Upon subsequent cooling to 50 °C, the micelles did not form an ordered structure and instead exhibited a liquid-like packing (LLP). When further cooled to 40 °C, the system was expected to organize into the σ phase according to the SAXS data collected during heating, yet it first formed a BCC phase, which subsequently transformed into the σ phase after 17.5 h of annealing at 30 °C, indicating that the BCC structure was metastable at lower temperatures. This ordering pathway was consistent with Ostwald’s step rule, which states that a system crystallizing from a supercooled liquid may first form a metastable structure whose free energy is closer to that of the liquid phase, rather than directly adopting the most stable structure.^37^ For BCP micelles, BCC frequently acted as a metastable precursor that subsequently evolved into the stable packing arrangement, such as the close-packed or FK phases observed here.^38,39^ This behavior also agreed with the Alexander–McTague mechanism, which predicts that a weakly first-order phase transition should favor the transient formation of a BCC phase before transformation into the stable lattice.^40^

Figure 2(a) displays the SAXS profiles of PEO-*b*-PI samples with varying PEO volume fractions at 30 °C, revealing a lyotropic phase transition from the BCC structure to the σ phase as *f*_*PEO*_ increased to approximately 0.25. Figure 2(b) displays the phase diagram of PEO-*b*-PI melts plotted in the χ*N*–*f*_*PEO*_ coordinates, based on the temperature-dependent SAXS results. The χ parameter describing the segmental incompatibility between the two blocks followed the relation χ = *A*/*T* + *B*, where *A* = 255 and *B* = −0.314, as reported by Willis et al.^41^ The degree of polymerization *N* was calculated based on a reference volume of 118 Å^3^ (see Table 1), using homopolymer densities of ρ_*PEO*_ = 1.21 g/cm^3^ and ρ_*PI*_ = 0.90 g/cm^3^.

**Figure 2:**
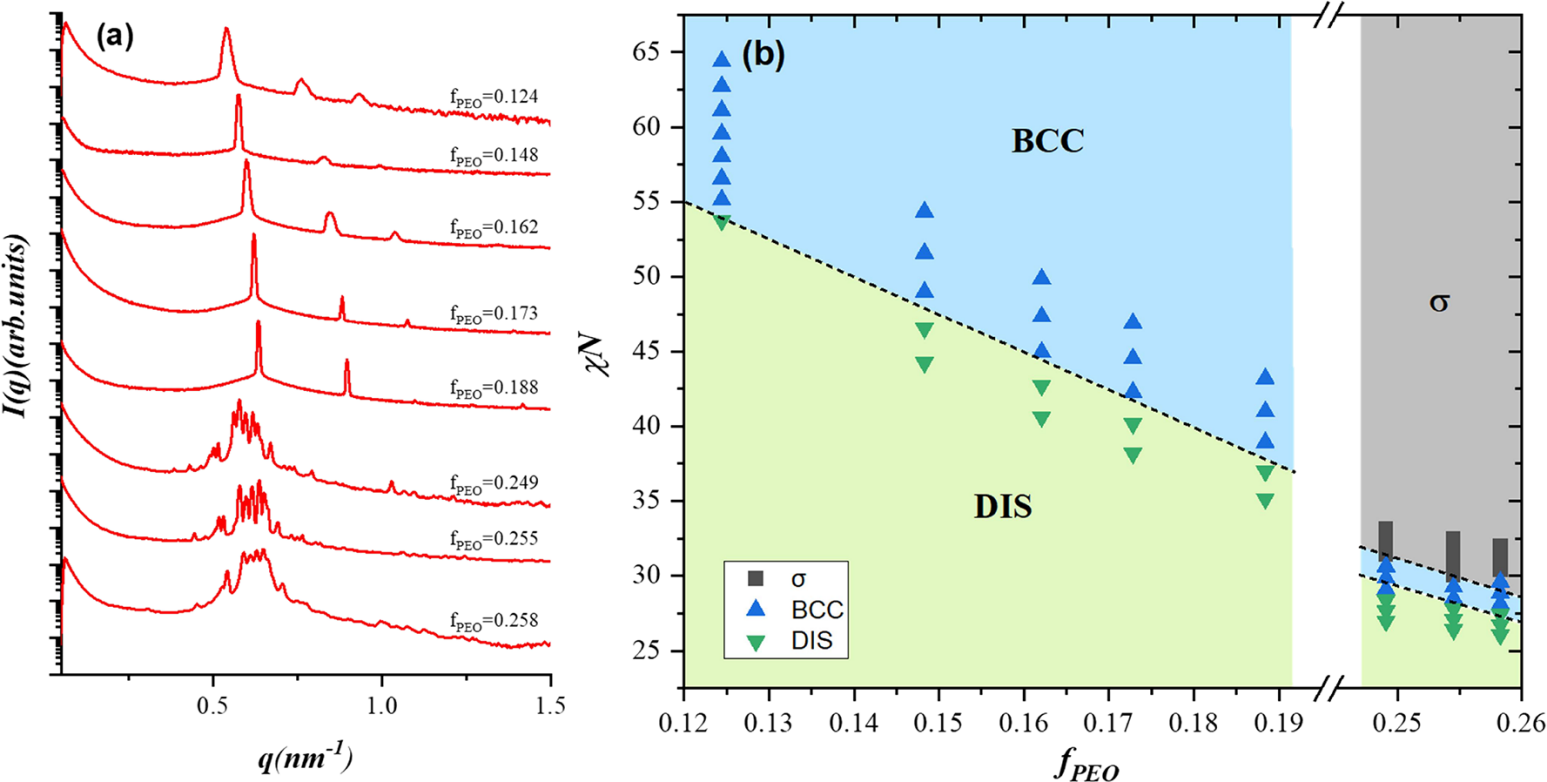
(a) SAXS profiles of PEO-*b*-PI with varying compositions at 30 °C, illustrating the transition from the BCC to the σ phase as *f_PEO_* increases to approximately 0.25. (b) Phase diagram of PEO-*b*-PI constructed from SAXS data obtained during the heating process. PEO-*b*-PI samples with *f_PEO_* < ∼0.19 formed the BCC phase, whereas those with *f_PEO_* ∼ 0.25 formed the FK σ phase. The σ phase in the PEO-*b*-PI system appeared at higher core volume fractions than in previously reported block copolymer systems.

Comparison among PI-*b*-PLA (ε = 1.15),^7^ PEE-*b*-PLA (ε = 1.30)^14^ and the present PEO-*b*-PI (ε = 1.26) systems shows that increasing ε led to a systematic expansion of the σ-phase region, accompanied by a compression of the BCC phase window. In the PEE-*b*-PLA, which has the highest ε, the σ phase even transitioned directly to the disordered state without passing through a BCC phase during heating.^14^ However, the SCFT calculation predicted that BCC phase always intervenes the σ-to-disorder phase transition.^13^ These differences may be ascribed to the mean-field nature of the SCFT, which neglects the fluctuation effects. While the mean-field theory predicted BCC stability at high temperature (low χ*N*), fluctuation effects in real systems may erase this narrow BCC basin at high ε, producing a direct transition from σ to disordered phase.

A particularly notable feature of the PEO-*b*-PI system is that the σ phase could form at a relatively high core volume fraction (*f*_*PEO*_ ∼ 0.26), whereas in PI-*b*-PLA and PEE*-b*-PLA, hexagonally packed cylinder (HEX) phase dominated at this core fraction.^14^ The sphere–cylinder boundary reflects the competition between interfacial free energy (*F*_*int*_) arising from the core–corona repulsion and conformational free energy (*F*_*conf*_). *F*_*int*_ is proportional to χ^1/2^ *N* ^1/3^ in the strongly segregated regime;^42^ thus, larger χ*N* favors cylindrical over spherical morphology at a fixed core volume fraction, as the tendency to alleviate the core–corona repulsion (and thus reduce *F*_*int*_) becomes more significant. Conversely, a higher *ε* promotes the formation of spherical phase at higher *f*_*core*_, in that relieving stretching or the conformational free energy of the majority block becomes dominant.

Among the three systems, PEO-*b*-PI exhibited the lowest *T*_*ODT*_, indicative of the weakest segregation strength, together with the second-highest ε value. This combination of a lower χ*N* and moderately high ε favored the stabilization of the σ phase at higher core volume fractions compared with previously reported systems.

### 3.2 Role of Intermicellar Overlap in Stabilizing Frank–Kasper Phases and the Micelle Size Expansion across the BCC−σ Transition

The phase diagram in Figure 2 confirms that BCC and σ phases represented the thermodynamically stable packing structures at low and high PEO core volume fractions (*f*_*c*_ = *f*_*PEO*_), respectively. The emergence of distinct packing lattices across different *f*_*c*_ regimes can be attributed to the varying extent of coronal chain confinement and intermicellar overlap that arise at the intermicellar boundaries under the constraint of melt incompressibility, which are elaborated in the following discussion.

In a spherical micelle, the coronal chains are densely tethered to the core surface. When the requirement of maintaining a uniform melt density within the corona is not imposed, the optimal chain conformation can be derived from the balance between the core–corona interfacial free energy and the chain conformational free energy. This balance yields an equilibrium coronal thickness of *l*_*cn0*_ ∼ χ^1/6^ *N*_*B*_^2/3^ *b*_*B*_ in the strong-segregation regime.^43^ Under these conditions, each coronal chain is predicted to extend radially outward while remaining confined within a conical sector, as schematically illustrated in Figure 3(a). Because the cross-sectional area of the core increases with radius, the local segmental density decreases correspondingly. As a result, chain segments adjacent to the core–corona interface experience higher entropic tension, whereas those near the outer corona are more relaxed. In the absence of the melt-density constraint, the corona would therefore expand to a thickness *l*_*cn0*_, at which the conformational free energy of the coronal chains is minimized under the influence of the interfacial repulsion.

**Figure 3:**
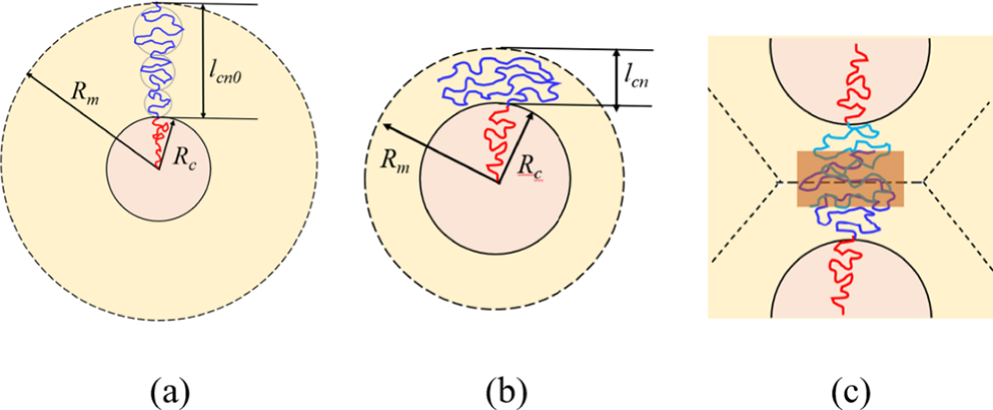
Schematic illustration of the coronal-chain conformations in spherical micelles under different confinement conditions. (a) In the absence of the melt-density constraint, each coronal chain extends radially from the core surface and occupies a conical sector, with its equilibrium coronal thickness (*l_cn0_*) determined by the balance between the core–corona interfacial free energy and the chain conformational free energy. (b) When the uniform melt-density constraint is imposed, the actual coronal thickness (*l_cn_*) becomes geometrically restricted by the core radius (*R_c_*) and the core volume fraction (*f_c_*), resulting in a thinner corona and chain extension preferentially along the tangential direction. (c) At elevated *f_c_* the confinement-induced strain in the corona can be partially relieved through intermicellar overlap or coronal-chain interdigitation between adjacent micelles, as indicated by the shaded region. Such interdigitation facilitates cooperative segmental interpenetration, maintaining melt incompressibility while reducing the elastic penalty associated with chain confinement.

In real systems, however, micelles must also satisfy the requirement of maintaining a uniform melt density. Under this constraint, the actual coronal thickness, *l*_*cn*_, is determined by the core radius, *R*_*c*_, and the core volume fraction, *f*_*c*_, as

1 $$l_{cn}=R_{c}\left(f_{c}^{-\frac{1}{3}}-1\right)$$

The geometric factor (*f*_*c*_^–1/3^ – 1) becomes smaller than unity when *f*_*c*_ > 0.125, indicating that the corona becomes thinner relative to the core radius at elevated *f*_*c*_, such that *l*_*cn*_/*l*_*cn0*_ ≪ 1. This pronounced thinning of the corona marks the regime in which FK phases are typically observed.

When the coronal thickness is reduced under the geometric constraint, the chains are confined within a narrower shell, forcing them to extend preferentially along the tangential direction rather than radially, as schematically illustrated in Figure 3(b). This geometrical confinement limits the chains’ ability to expand laterally and relax their conformational strain, leading to an increase in the overall conformational free energy of the coronal block. However, this confinement-induced strain can be partially relieved if the coronal chains intrude into the coronas of the adjacent micelles, forming intermicellar overlap or coronal-chain interdigitation, as illustrated in Figure 3(c). Such interdigitation restores conformational freedom and lowers the elastic penalty associated with confinement, while simultaneously maintaining melt incompressibility through cooperative segmental interpenetration between adjacent coronas.

At small core volume fractions, where the geometric factor (*f*_*c*_^–1/3^ – 1) is close to or greater than unity, the coronal chains experience only minor confinement along their trajectories. Consequently, overlap between adjacent coronas remains limited. Under the space-filling constraint, the micelles deform toward the shapes of their Voronoi cells, leading to a degree of nonuniformity in the coronal thickness. Although this nonuniformity could in principle be reduced if the cores were to deform into polyhedral shapes resembling the Voronoi cells, such deformation is inefficient when the cores are small, because the core–corona interface lies far from the micelle edges and is therefore only weakly coupled to the outer geometry. As a result, the cores remain nearly spherical at low *f*_*c*_. Since overlap between neighboring coronas is minimal and core deformation cannot effectively equalize the corona thickness, the uniformity of the coronal layer becomes the dominant factor governing lattice selection. The BCC lattice best satisfies this requirement, as its truncated octahedral Voronoi geometry provides the most uniform coronal thickness among all space-filling arrangements. The BCC phase is therefore the thermodynamically favored packing structure at low *f*_*c*_, where the total free energy is governed primarily by the conformational entropy of the coronal chains.

At elevated core volume fractions, the corona becomes significantly thinner relative to the optimum thickness, i.e., *l*_*cn*_/*l*_*cn0*_ ≪ 1, leading to strong confinement of the coronal chains. To relieve this compression, intermicellar overlap can occur, allowing partial interdigitation of coronal segments across neighboring micelles. While such overlap relaxes chain confinement and restores some conformational entropy, it simultaneously introduces orientational ordering of the overlapping segments, resulting in a loss of segmental orientational entropy.^44^ The total free energy at elevated *f*_*c*_ thus reflects a balance between these two competing effects: relief of confinement and the entropic penalty associated with segmental alignment within the overlap regions.

In this regime, complex FK lattices become thermodynamically favored because the polyhedral geometries of their Voronoi cells minimize the total intermicellar boundary area relative to that in the BCC lattice,^3,4^ thereby reducing the overall extent of coronal-chain overlap and the associated loss of segmental orientational entropy. Moreover, the contact zones are partitioned into smaller facets and edges, where localized chain splay can further alleviate the confinement-induced entropic penalty.

As the cores grow larger, deformation of the core toward the corresponding Voronoi cell geometry further promotes homogenization of the corona thickness, thereby diminishing the conformational free energy penalty associated with nonuniform chain dimension.^1,6^ Although such core deformation increases the overall core–corona interfacial area, the intrinsically lower surface area per unit volume characteristic of FK lattices partially compensates for this cost. Consequently, FK phases represent an optimized packing state that balances coronal confinement, intermicellar overlap, and interfacial free energy more effectively than the uniform BCC lattice, leading to their thermodynamic stabilization at elevated core volume fractions.

Figure 4(a) presents the variation of average micelle volume (⟨*V*_*m*_⟩) at 30 °C, along with the respective volumes of the PEO core (*V*_*c*_) and PI corona (*V*_*cn*_), plotted as a function of *f*_*PEO*_. Within the BCC region, both *V*_*m*_ and *V*_*cn*_ decreased monotonically with increasing *f*_*PEO*_, consistent with the progressive reduction of PI molecular weight, whereas *V*_*c*_ remained nearly constant since the PEO block length was fixed. Strikingly, all three volumes exhibited a sudden increase as the lattice transformed from the BCC to the σ phase, despite the continued decrease in PI molecular weight. Beyond this transition, *V*_*m*_ and *V*_*cn*_ again decreased with further increase in *f*_*PEO*_.

**Figure 4:**
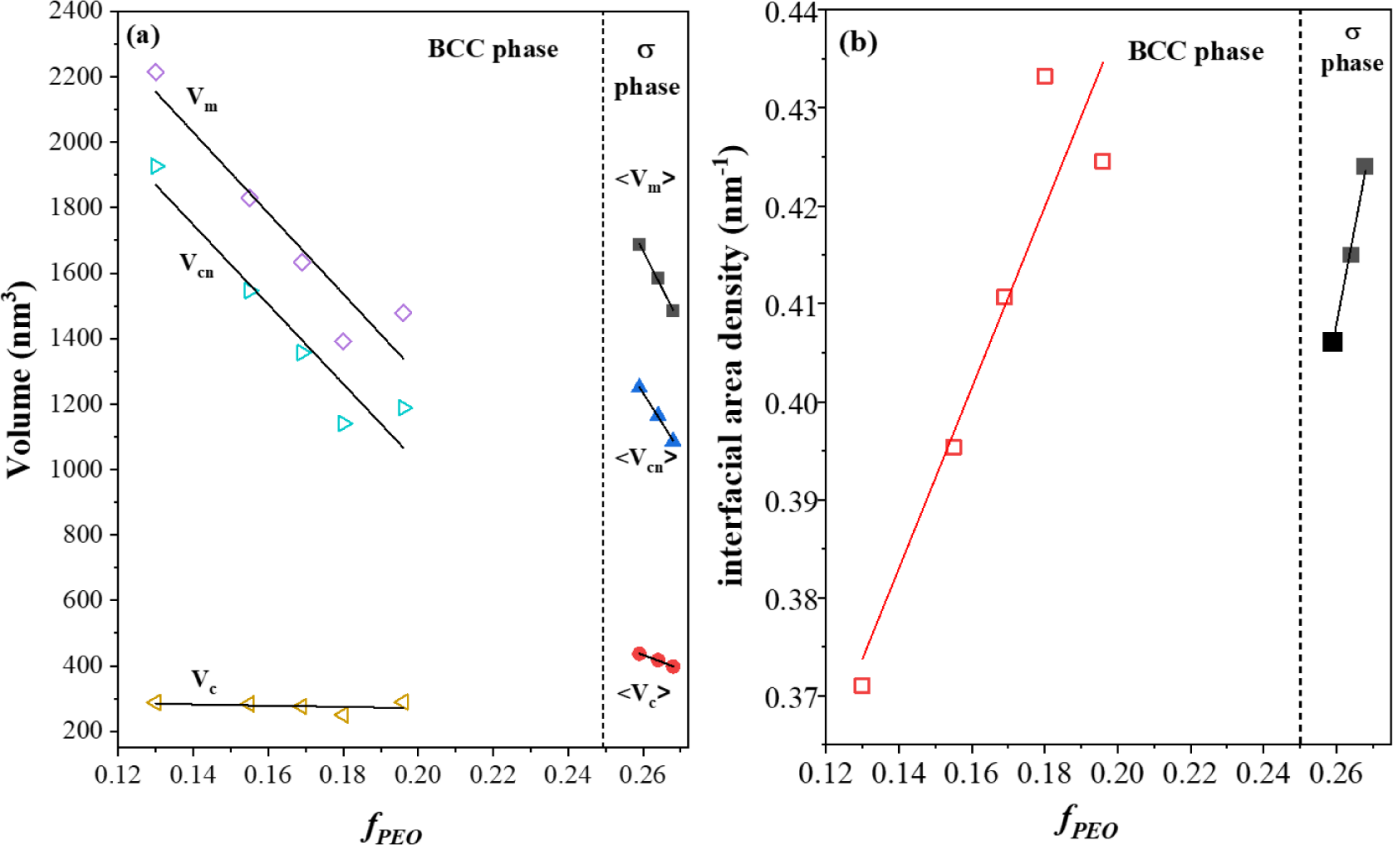
Variations of (a) the average micelle volume (⟨*V*_m_⟩) at 30 °C, along with the corresponding volumes of the PEO core (*V_c_*) and PI corona (*V_cn_*), and (b) the interfacial area density as a function of *f_PEO_*. Within the BCC phase region, both *V_m_* and *V_cn_* decrease monotonically with increasing *f_PEO_*, reflecting the progressive reduction in PI molecular weight. At the transition to the σ phase, all three volumes exhibit a pronounced increase despite the continued decrease in PI molecular weight. This abrupt micelle expansion signifies a reorganization of the free energy balance as the packing symmetry shifts from the single-site BCC lattice to the multisite σ lattice, which is further manifested by the concurrent reduction in interfacial area density.

This abrupt micelle expansion signals a reorganization of the free energy balance when the packing symmetry changed from the single-site BCC lattice to the multisite FK σ lattice. In the BCC structure, which contains only one type of Voronoi cell, the optimal micelle size is determined by the intramicellar balance between the stretching free energy of the coronal chains, which favors smaller micelles, and the interfacial energy between the core and corona, which favors larger micelles. Transitioning to the σ lattice alters this balance through three geometrical and entropic effects: (i) the average contact area per micelle is reduced, (ii) the presence of inequivalent cells allows polydisperse adjustments in micelle size, and (iii) anisotropic chain stretching becomes more pronounced. Because the intermicellar overlap in the σ phase reduces the segmental orientational entropy, effectively producing an entropic repulsion that drives the system to minimize the interfacial contact area between micelles. The σ lattice accommodates this tendency by partitioning the intermicellar interfaces into smaller facets separated by edges and vertices, thus lowering the total interfacial area per micelle and reducing the corresponding overlap-related free energy penalty.

At high core volume fractions and low PI block molecular weights, this geometric advantage becomes increasingly significant. The entropic gain from reducing overlap and associated entropy loss outweighs the additional cost of nonuniform chain stretching, thereby shifting the free energy minimum toward a larger micelle dimension. The sudden increase in micelle volume is a hallmark of FK phases in BCP systems, reflecting a thermodynamic readjustment accompanying the transition from the uniform BCC lattice to the geometrically complex σ lattice. This readjustment is driven by the need to minimize the overall interfacial and overlap free energy penalties. Consistent with this interpretation, Figure 4(b) shows that the interfacial area density of the micelles exhibits a distinct decrease at the BCC-to-σ transition, providing quantitative support for the reduction of interfacial contributions upon formation of the σ phase.

### 3.3 Phase Behavior of the Blend of PEO-*b*-PI with h-PEO in the Dry-Brush Regime

To enlarge the stability window of FK phases, we blended an h-PEO (*M*_*h‑PEO*_ ≈ 1300 g/mol, corresponding to the value of α = *M*_*h‑PEO*_/*M*_*b‑PEO*_ ≈ 1) into PEO-*b*-PI. The design principle was to promote dry-brush mixing of h-PEO with the PEO block (b-PEO), thereby enabling spherical packings at even higher overall PEO volume fraction. Blends were prepared using the BCC-forming PEO^1.3^-*b*-PI^4.5^ (*f*_*PEO*_ = 0.19).

The dry-brush behavior in the PEO-*b*-PI/h-PEO blends was supported by examining the melting behavior of the PEO components forming the micellar cores. As shown in Figure S5, the DSC heating thermograms of the blends crystallized upon cooling to −30 °C exhibited two distinct melting endotherms: a lower-temperature peak associated with the melting of the PEO block and a higher-temperature peak corresponding to the melting of h-PEO. The absence of a single, merged melting transition indicates that the PEO blocks and h-PEO did not cocrystallize or mix homogeneously at the molecular level. Instead, h-PEO remained spatially segregated within the PEO microdomain, consistent with dry-brush behavior and giving rise to a three-layer micellar architecture comprising an h-PEO core, a b-PEO-enriched shell, and a PI corona.

Figure 5(a) presents the temperature-dependent SAXS profiles of the PEO^1.3^ *-b*-PI^4.5^/h-PEO blend with the overall h-PEO volume fraction *f*_*h‑PEO*_ = 0.03, which increased *f*_*PEO*_ to 0.21. A small peak was observed beside a broad principal peak as marked by the arrow. This scattering pattern, having been observed previously, was assigned as a “quasi-σ phase (Q-σ)”.^23^ In this assignment, the weaker peak corresponds to the (002) reflection arising from the layer periodicity along the tetragonal *c*-axis. The lack of additional characteristic reflections, expected to fall within the breadth of the principal maximum, indicates poor in-plane (lateral) order of micelles within each layer.

**Figure 5:**
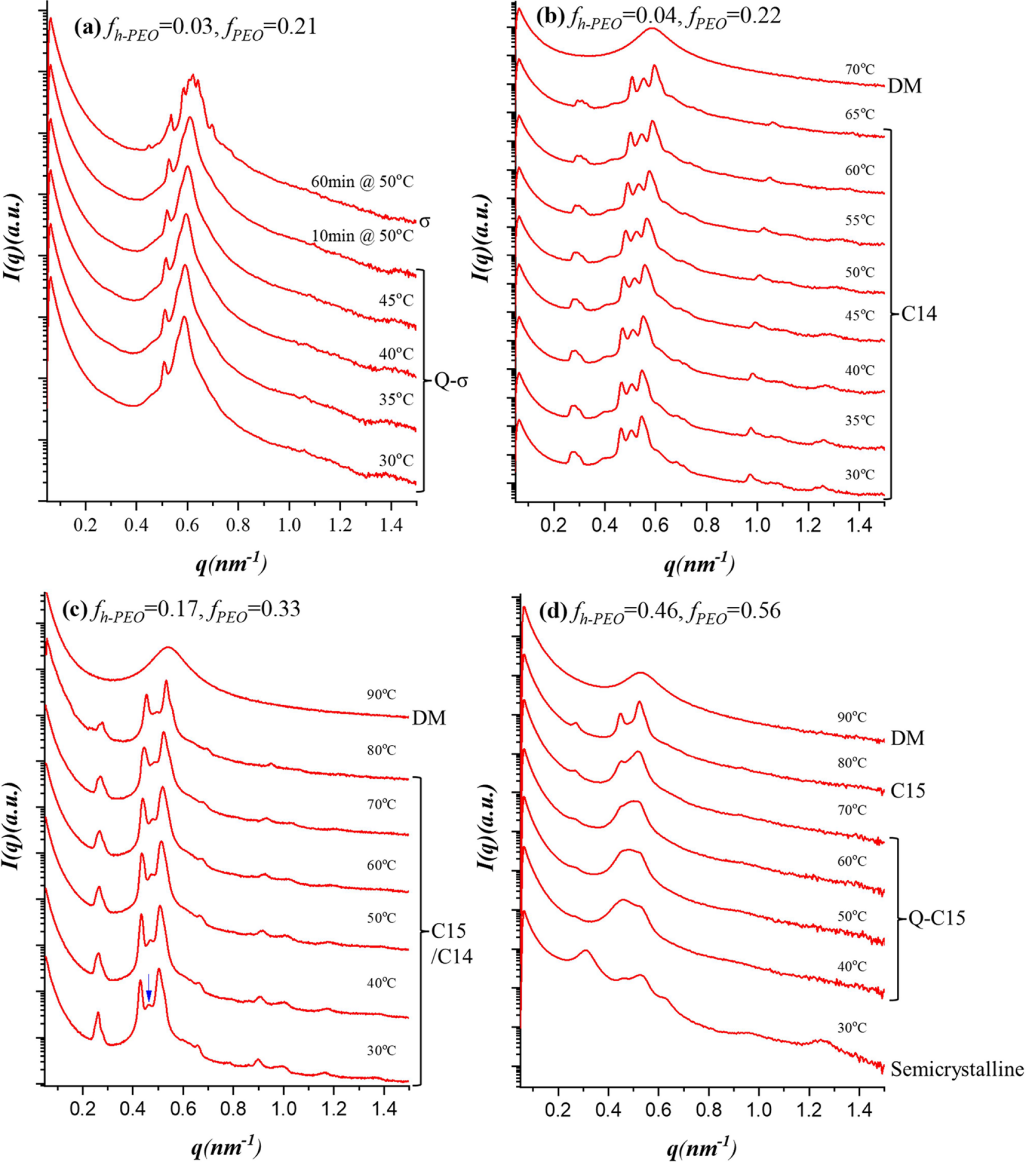
Temperature-dependent SAXS profiles of PEO^1.3^-*b*-PI^4.5^/h-PEO blends with (a) *f_PEO_* = 0.21, (b) *f_PEO_* = 0.22, (c) *f_PEO_* = 0.33 and (d) *f_PEO_* = 0.56, collected during heating. The structural phases identified at various temperature ranges are labeled in the figure. In (a), a quasi-σ (Q-σ) phase characterized by incomplete lateral ordering transformed into the σ phase upon prolonged annealing at 50 °C. Increasing the h-PEO content to *f_h‑PEO_* = 0.04 produced the Laves C14 phase (b), while further addition (*f_h‑PEO_* = 0.17) yielded the C15 phase (c), with a weak residual feature indicating coexistence of C14. At the highest h-PEO loading (*f_h‑PEO_* = 0.46; *f_PEO_* = 0.56), macrophase separation occurred, and the unconfined h-PEO crystallized at 30 °C, forming a semicrystalline state. Upon heating, the system evolved through a quasi-C15 (Q-C15) state and ultimately reorganized into the C15 phase near 80 °C. Collectively, the series reveals a sequential structural evolution of the blends: from the σ phase to C14, then to C15, and ultimately to macrophase separation, as the h-PEO content increased.

Despite annealing at room temperature for several days, the Q-σ phase persisted as a long-lived metastable morphology, reflecting sluggish lateral ordering at low temperature. To promote further structural relaxation, the sample was subsequently annealed isothermally at 50 °C. After 10 min at 50 °C, the SAXS pattern remained characteristic of the Q-σ state. In contrast, after 60 min of annealing, multiple well-defined reflections developed and could be unambiguously indexed to the σ phase (Figure S6 in the SI). These observations demonstrate that the Q-σ structure corresponded to a kinetically trapped state that arose from insufficient lateral ordering at low temperature and evolved into the equilibrium σ phase upon prolonged annealing at elevated temperature.

Figure 5(b) shows the SAXS profiles of the blend with *f*_*h‑PEO*_ = 0.04 (*f*_*PEO*_ = 0.22). The pattern matched the Laves C14 phase (*P*6_3_/*mmc*) with the hexagonal lattice parameters *a* = 26.95 nm and *c* = 44.23 nm, as demonstrated in Figure S7­(a) in SI. Upon increasing the h-PEO content to *f*_*h‑PEO*_ = 0.17 (*f*_*PEO*_ = 0.33), the SAXS pattern (Figure 5(c)) evolved into one that was consistent with the Laves C15 phase (Fd3̅m), characterized by a cubic lattice parameter *a* = 39.94 nm (Figure S7­(b) in the SI). In addition to the dominant C15 reflections, a weak feature at *q* ≈ 0.50 nm^– 1^ (pinpointed by the blue arrow) was observed, which can be assigned to the C14 (103) reflection. We therefore described this structure as a C15/C14 coexistence, in which the C15 lattice was dominant while a minor C14 contribution was present.

The presence of weak C14 features within the nominal C15 region can be attributed to the very small free energy difference between the two Laves structures. SCFT calculations conducted by Xie and Shi evaluated the total free energy among different FK phases in BCP/homopolymer blends under the conditions of ε = 1.25, α = 1, and χ_*AB*_ = 0.45, which are comparable to our experimental conditions. The results showed that the C15 phase exhibited a slightly lower Helmholtz free energy density than the C14 phase when the homopolymer volume fraction exceeded approximately 0.04, but the difference between the two phases remained minimal.^35^ Importantly, the presence of a minor C14 phase coexisting with a predominantly C15 structure did not necessarily indicate that the C14 phase was metastable. Laves phases are polytypic structures whose crystallographic identities (C14, C15, and C36) are determined by the stacking sequence of structurally equivalent two-dimensional layers.^45^ When the free energy difference between competing stacking sequences is small, as predicted by SCFT in this composition range, finite grain size and thermal fluctuations may give rise to an equilibrium degree of stacking disorder or mixed polytypism. Under such conditions, local C14-type stacking motifs may coexist within a majority C15 lattice, leading to the simultaneous observation of scattering features associated with both structures. Similar behavior has been reported previously for close-packed micellar phases, where finite grain-size effects rendered the entropy of stacking-sequence mixing non-negligible and resulted in equilibrium stacking faults.^46,47^

Finally, Figure 5(d) shows the SAXS profiles of the PEO^1.3^-*b*-PI^4.5^/h-PEO blend with *f*_*h‑PEO*_ = 0.46, corresponding to *f*_*PEO*_ = 0.56. The excessive addition of h-PEO exceeded the capacity of the micelle core, leading to macrophase separation. At 30 °C, the unconfined h-PEO underwent crystallization, as evidenced by the corresponding WAXS profile in Figure S4, which disrupted the long-range order of the system, resulting in a semicrystalline state characterized by a scattering pattern with broad peaks. The h-PEO crystallites melted at approximately 40 °C; however, at this temperature the micelles had not yet reorganized into an ordered lattice. Under these conditions, a weak low-*q* peak at *q* ≈ 0.26 nm^–1^ indicated the emergence of local micellar order resembling that of the Laves phase. Upon further annealing at elevated temperature, a well-developed C15 phase was eventually formed at 80 °C. Accordingly, this intermediate packing state observed at lower temperatures was referred to as a quasi-C15 (Q-C15) phase, representing a kinetically trapped state that preceded the formation of the equilibrium C15 lattice.

Figure 6 presents the morphological diagram of the PEO^1.3^-*b*-PI^4.5^/h-PEO blends, constructed based on the SAXS results. Along the composition axis, the blends exhibited a lyotropic sequence of spherical packing transitions of BCC → σ → C14 → C15 with increasing *f*_*h‑PEO*_. This sequence was consistent with theoretical predictions by Xie and Shi, who employed SCFT with a freely jointed chain model to investigate the relative stabilities of spherical phases in A-*b*-B/h-A blends under dry-brush conditions.^35^ Among the four considered FK phases (σ, A15, C14, and C15), the σ phase was found to be entropically favored at low homopolymer contents, exhibiting the lowest copolymer block–block interaction energy and thus the greatest thermodynamic stability. However, as the homopolymer content increased, the h-A/B block interaction energy in the Laves phases (C14 and C15) decreased markedly, thereby stabilizing these phases at higher compositions.

**Figure 6:**
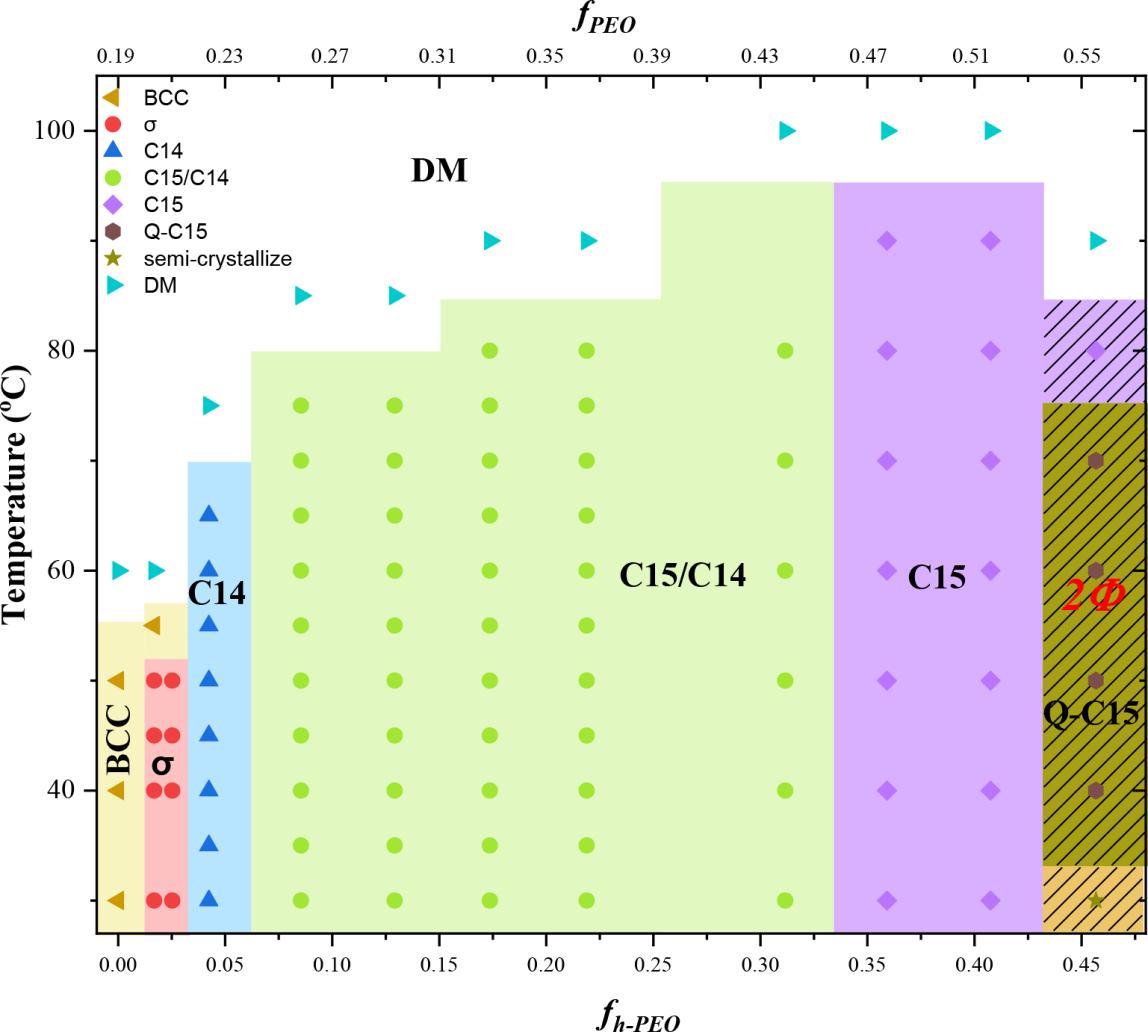
Morphological diagram of PEO^1.3^-*b*-PI^4.5^/h-PEO dry-brush blends, constructed from the temperature-dependent SAXS results obtained during the heating process. The diagram illustrates the sequential structural evolution of the blends, from the BCC phase to the σ phase, followed by the Laves C14 phase, a C15/C14 coexistence region, and finally the C15 phase, as the h-PEO content increases. The hatched region denotes the macrophase-separated regime, in which excess h-PEO crystallizes below its melting temperature. In this regime, the local composition within the microphase-separated domains may deviate from the nominal blend composition; nevertheless, the nominal composition is used consistently in constructing the morphological diagram to provide a clear and reproducible representation of the experimental conditions.

Such a BCC−σ–C14–C15 transition sequence has also been reported in other dry-brush blend systems. Mueller and coworkers showed that blending a BCC-forming polystyrene-*block*-poly­(1,4-butadiene) (PS-*b*-PB) with h-PB led to sequential access to the σ, C14, and C15 phases with increasing h-PB content, albeit at different temperatures.^32^ Complementary SCFT calculations further demonstrated that, even at constant χ*N*, increasing the homopolymer volume fraction alone can drive this sequence of spherical packing transitions.^35^ This theoretical prediction was subsequently validated experimentally by Mueller et al. in poly­(ethylene oxide)-*block*-poly­(2-ethylhexyl acrylate) (PEO-*b*-PEHA)/h-PEO blends with different homopolymer molecular weights.^34^ The agreement between our experimental observations and these theoretical and experimental precedents highlights the generality of homopolymer-induced lyotropic transitions among spherical packings in dry-brush BCP/homopolymer blends.

It is worth noting that the FK phase region in the present PEO^1.3^-*b*-PI^4.5^/h-PEO blend system spanned an unusually broad composition range compared to previously reported BCP/homopolymer blends. As shown in Figure 6, the FK phases persisted up to an overall PEO volume fraction of approximately 0.5, corresponding to homopolymer fractions as high as *f*_*h‑PEO*_ ≈ 0.4, before macrophase separation occurred. This is significantly higher than the homopolymer contents at which macrophase separation typically occurred in other systems, where the stability window for FK phases was often restricted to relatively narrow composition ranges.^32,34^ The origin of this unusually wide FK phase stability window is to be analyzed in the following section.

### 3.4 Composition-Dependent Variation of Micellar Structural Parameters in PEO-*b*-PB/h-PEO Dry-Brush Blends

The incorporation of h-PEO into the PEO microdomain induced systematic and nonmonotonic variations in the micellar structural parameters, as shown in Figure 7, which summarizes their dependence on *f*_*h‑PEO*_ at 30 °C. Owing to the dry-brush mixing behavior between b-PEO and h-PEO, each micelle adopted a three-layer architecture comprising an h-PEO core, a b-PEO shell, and a PI corona. To quantify the structural evolution, we first calculated the average micelle volume and subsequently determined the volumes of the three microphases by multiplying the micelle volume by their respective volume fractions, assuming that all added h-PEO was incorporated into the PEO microdomain and that complete segregation was achieved. The detailed procedures and equations used to derive these structural parameters are provided in Section 6 of the SI.

**Figure 7:**
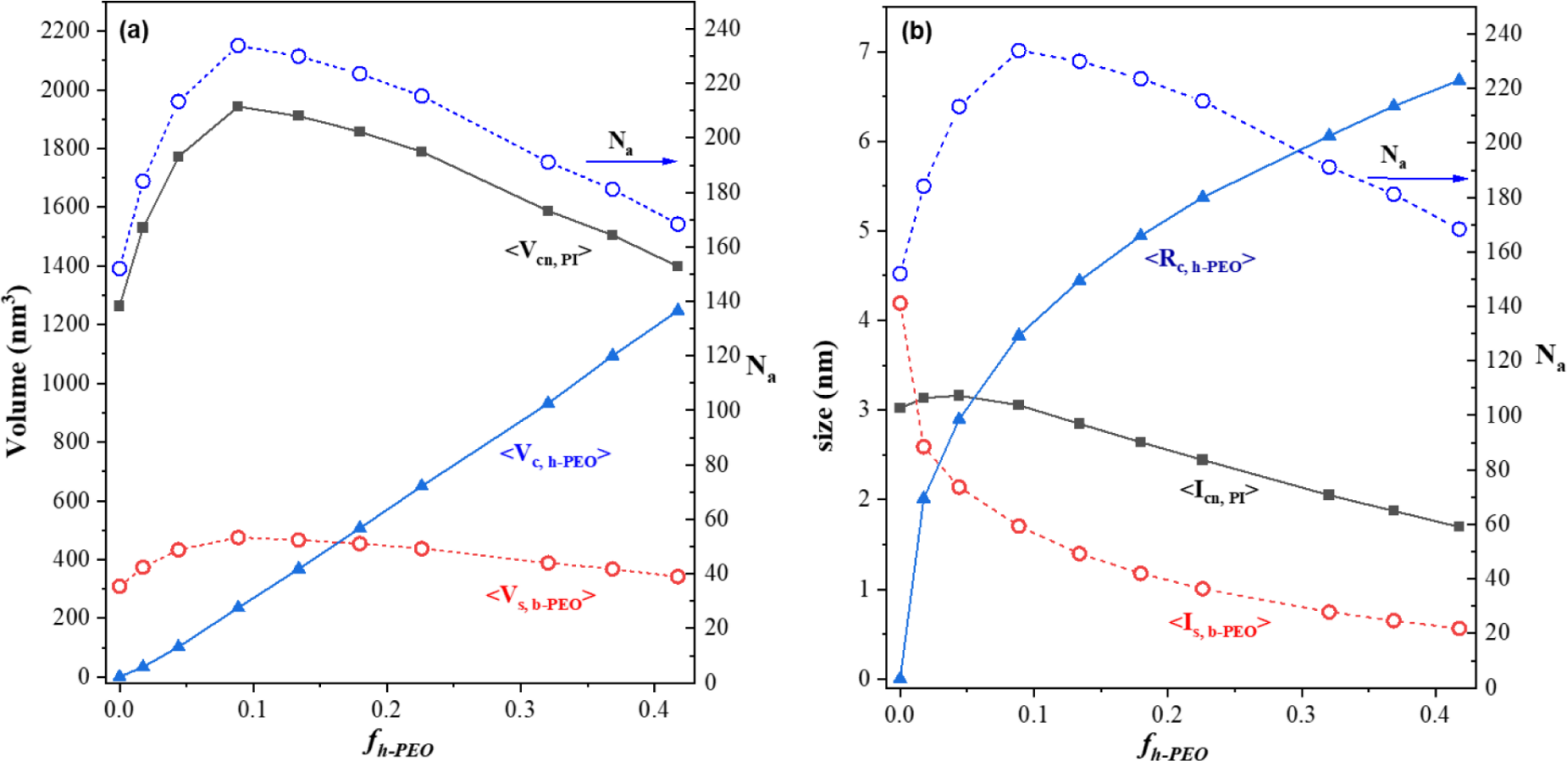
(a) Average volumes of the PI corona (⟨*V_cn,PI_* ⟩), b-PEO shell (⟨*V_s,b‑PEO_* ⟩), and h-PEO core (⟨*V_c,h‑PEO_* ⟩), together with the micelle association number (*N_a_*), plotted as a function of h-PEO volume fraction (*f_h‑PEO_*). (b) Corresponding characteristic dimensions, including the PI corona thickness (⟨*l_cn,PI_* ⟩), b-PEO shell thickness (⟨*l_s,b‑PEO_* ⟩), and h-PEO core radius (⟨*R_c,h‑PEO_* ⟩). At low *f_h‑PEO_*, both *N_a_* and micelle volume increased as the h-PEO chains filled the cores, slightly expanding the PI corona while thinning the b-PEO shell. Beyond this regime, further addition of h-PEO caused *N_a_* to decrease, accompanied by continuous growth of the h-PEO core and progressive thinning of the PEO and PI layers. This trend reflects the system’s effort to relieve chain confinement and interfacial compression, leading to enhanced interdigitation between adjacent micelles and a thermodynamic driving force favoring a transition from the σ to Laves phase at higher h-PEO contents.

Figure 7(a) shows how the association number (*N*_*a*_) and the volumes of the PI corona (*V*_*cn,PI*_), b-PEO shell (*V*_*s,b‑PEO*_), and h-PEO core (*V*_*c,h‑PEO*_) varied with *f*_*h‑PEO*_; *N*_*a*_ was determined from *V*_*cn,PI*_ using the relation

2 $$N_{a}=\frac{V_{cn,PI}\rho_{PI}N_{av}}{M_{b‐\mathrm{PI}}}$$

where ρ_*PI*_ and *M*_*b‑PI*_ are the mass density and molecular weight of PI block, respectively, and *N*_*av*_ is the Avogadro’s number.

Next, each micelle was modeled as an equivalent sphere in which the three microphases occupied the same volumes as in the actual (deformed) structure. This approximation enabled the calculation of key structural parameters, including the PI corona thickness (*l*_*cn,PI*_), b-PEO shell thickness (*l*_*s,b‑PEO*_), and the h-PEO core radius (*R*_*c,h‑PEO*_) (see Section 6 of SI). The dependence of these characteristic sizes on *f*_*h‑PEO*_ is presented in Figure 7(b).

We would like to emphasize that the equivalent-sphere construction used in Figure 7 was purely geometric in nature and was not intended as a thermodynamic model for FK phase stability. This representation served only as a convenient visualization tool to track how material redistributed among the h-PEO core, b-PEO shell, and PI corona as the h-PEO fraction increased. In FK phases, micelles occupy inequivalent polyhedral Voronoi cells whose volumes, surface curvatures, and number densities differ across BCC, σ, C14, and C15 lattices. Consequently, the free energy density could not be inferred by comparing the volumes of equivalent spheres. As established in the diblock foam picture and SCFT, the thermodynamic selection of FK lattices arises from a balance between polyhedral interfacial area and heterogeneous chain deformation, features that are inherently absent in an equal-volume sphere approximation. Accordingly, Figure 7 should be interpreted solely as a qualitative geometric description of micellar restructuring with increasing h-PEO content, rather than as a basis for semiquantitative free energy comparison or lattice ranking.

As shown in Figure 7(a), at low h-PEO volume fractions (*f*_*h‑PEO*_ < ∼0.1), *N*_*a*_, *V*_*cn,PI*_, and *V*_*s,b‑PEO*_ increased with increasing h-PEO content. In this regime, the PI corona thickness increased slightly, whereas the b-PEO shell thickness decreased markedly (Figure 7(b)) despite the increase in its overall volume (Figure 7(a)). When only a small amount of h-PEO was added, the h-PEO cores within the micelles were very small, imposing a strong confinement entropy penalty on the h-PEO chains due to restricted conformational degree of freedom. To alleviate this unfavorable entropic cost, the system reduced the micelle number density through increasing the association number, allowing each micelle to accommodate a larger h-PEO core. This enlargement of the core slightly expanded the PI corona thickness, while the b-PEO shell thinned due to the increased core surface area that must be covered by a fixed number of b-PEO chains. Consequently, even though the volume of the b-PEO shell increased, its thickness decreased monotonically with increasing *f*_*h‑PEO*_ in this low-*f*_*h‑PEO*_ regime.

As *f*_*h‑PEO*_ approached approximately 0.1, both the association number and PI corona thickness reached a maximum. Beyond this point, further addition of h-PEO led to a decrease in *N*_*a*_, accompanied by continuous growth of the h-PEO core radius and progressive thinning of both the PI corona and PEO shell. This evolution signified a shift in the dominant free energy contributions governing micelle packing. If the micelles were to retain a large association number at elevated h-PEO contents, the h-PEO cores would become excessively large, imposing severe packing constraints on the surrounding PEO and PI layers. As the interfacial area per chain increased more rapidly than the corona volume per chain, the PI and b-PEO layer thicknesses shrunk significantly, leading to pronounced compression and confinement of both the PI and b-PEO chains.

To alleviate this confinement, the system reduced *N*_*a*_ and redistributed the added h-PEO among a larger number of micelles to moderate the core growth. In addition, partial interpenetration of the b-PEO chains into the h-PEO core formed an interdigitated layer that relaxed their local chain compression, while the PI coronas of neighboring micelles partially overlapped. These relaxation mechanisms, however, incurred an orientational entropy loss for the overlapping segments. As the degree of interdigitation increased with h-PEO content, the resulting orientational entropy penalty grew, creating a strong thermodynamic driving force to minimize the interfacial area per micelle. Consequently, the system transitioned from the σ phase to a Laves phase at higher h-PEO compositions, as the Voronoi geometry of Laves lattices offered an even smaller total interfacial area^3,4^ and thus mitigated the entropic cost of interdigitation. Importantly, this transition was not inferred from the equivalent-sphere construction itself, but rather from the well-established geometric property that Laves lattices provide a smaller total interfacial area per micelle than the σ phase, thereby more effectively reducing the entropic penalty associated with increased interdigitation at high h-PEO contents.

Nevertheless, the micelles in the Laves phase are more volume-asymmetric than those in the σ phase, leading to a higher conformational entropy penalty.^3^ At *f*_*h‑PEO*_ ≳ 0.4, the PI and b-PEO chains became too compressed, strongly resisting further incorporation of h-PEO into the microdomains. At this point, the free energy cost of confinement and interdigitation outweighed the driving force of h-PEO incorporation, resulting in the onset of macrophase separation.

The overall composition dependence of micelle structure reflected a delicate balance among three competing factors: (i) the confinement entropy penalty of h-PEO chains, which favored larger micelles and a lower micelle number density at low h-PEO contents; (ii) the elastic penalty associated with the confinement and compression of the PEO and PI blocks at large core sizes, which drove a reduction in association number at higher h-PEO contents; and (iii) the incompressibility constraint, which enforced the redistribution of h-PEO through structural reorganization of the micelles. This interplay gave rise to the nonmonotonic dependence of *N*_*a*_ and corona dimensions on h-PEO composition and underscored the critical role of chain confinement in limiting core swelling in BCP/homopolymer blends. Figure S8 provides a schematic summary of the micellar structural evolution and the competition among these entropic contributions as *f*_*h‑PEO*_ increased.

### 3.5 Stabilization of the Spherical Phase over a Broad Composition Window

The morphological diagram in Figure 6 revealed that the spherical phase with C15 packing remained stable even at *f*_*PEO*_ ≈ 0.5, close to a symmetric composition, beyond which macrophase separation occurred. This behavior contrasted sharply with previous reports,^32,34^ where phase separation typically emerged at substantially lower homopolymer contents, highlighting the unusually broad stability window of the spherical phase in the present blend system. The elevated saturation composition of h-PEO accommodated within the PEO microdomains can be rationalized by the influence of chain length on the competing entropic penalties that govern micelle swelling. In this system, both the PEO-*b*-PI and h-PEO components possessed comparatively low molecular weights. First, the confinement penalty associated with incorporating h-PEO chains into the micelle core decreased with shorter chain length, as smaller coils experienced less entropic loss under a given spatial constraint. This facilitated core expansion before the system incurred substantial free energy penalties. Second, the elastic penalties that limited further swelling arose when the b-PEO and PI chains became strongly compressed or confined. Saturation occurred when the corona layers thinned to a fraction of their unperturbed coil dimensions, beyond which the elastic free energy per chain increased steeply. For short BCP chains, the natural coil size is smaller, allowing the corona and interfacial layers to accommodate greater absolute thinning before reaching this compression threshold. As a result, more h-PEO can be incorporated before the onset of prohibitive confinement.

Additionally, a smaller overall degree of polymerization reduced χ*N* value, softening both interfacial and interdigitation penalties within the dry-brush PEO region and further facilitating h-PEO uptake. Collectively, the reduced confinement cost for short h-PEO chains and the greater allowable thinning of the short b-PEO and PI layers acted synergistically to shift the saturation limit toward higher homopolymer compositions, thereby stabilizing the spherical phase over an unusually broad composition range.

## 4 Conclusions

This study systematically investigates the phase behavior of PEO-*b*-PI in the spherical packing regime and identifies it as a new linear diblock copolymer system capable of forming FK phases. Owing to its relatively low χ*N* and moderate conformational asymmetry (ε ≈ 1.26), the σ phase appeared at a higher core volume fraction than in previously reported systems. Notably, in the neat PEO-*b*-PI system, we observed an abrupt enlargement of micelle size across the BCC-to-σ lattice transition, despite a reduction in molecular weight. This striking size change underscored the pronounced influence of lattice symmetry on micelle dimensions at the onset of FK phase formation.

To further extend the accessible FK phase regime, we employed a core dry-brush blending strategy (α = 1) by incorporating h-PEO into a BCC-forming PEO-*b*-PI. The blends followed a lyotropic BCC → σ → C14 → C15 phase transition sequence that was broadly consistent with SCFT predictions. A key outcome of this study was the unusually broad composition window for FK phase stability in the blends, which allowed the well-ordered Laves C15 phase to form at nearly symmetric compositions without macrophase separation. This behavior can be rationalized by chain-length effects on the entropic penalties that governed the micelle swelling. The relatively low molecular weights of both the BCP blocks and h-PEO reduced the core confinement penalty, allowed greater thinning of corona layers before reaching critical compression, and softened interfacial penalties due to lower χ*N*. Together, these factors shifted the saturation limit toward higher homopolymer compositions.

The micelle structural parameters of the blends exhibited systematic and nonmonotonic variations with increasing h-PEO content. At low h-PEO fractions (*f*_*h‑PEO*_ < 0.1), the association number and micelle volume increased to relieve homopolymer confinement, leading to PI corona expansion and b-PEO shell thinning. At higher h-PEO contents, the association number decreased while the h-PEO core continued to grow and both corona and shell thinned progressively, reflecting the increasing role of chain compression penalties. At *f*_*h‑PEO*_ ≳ 0.4, PI and PEO block chains became highly compressed, resisting further swelling and defining the saturation limit.

Overall, this work establishes PEO-*b*-PI and its dry-brush blends as a chemically simple yet versatile model system for stabilizing FK phases. By linking lattice symmetry effects, homopolymer-induced micelle restructuring, and chain length-dependent free energy penalties, this study adds a physical framework for understanding and designing BCP systems with expanded stability windows for complex spherical phases.

## Supplementary Material



## Notes

Synthesis and characterizations of PEO-*b*-PI; wide-angle X-ray scattering (WAXS) profiles of neat PEO-*b*-PI and PEO-*b*-PI/h-PEO dry-brush blends equilibrated at 30 °C; differential scanning calorimetry (DSC) evidence for dry-brush segregation in PEO-*b*-PI/h-PEO blend; SAXS peaks of the PEO^1.3^-*b*-PI^4.5^/h-PEO blend with *f*_*h‑PEO*_ = 0.03, indexed to the characteristic reflections of the Frank–Kasper σ phase; SAXS peaks of the PEO^1.3^-*b*-PI^4.5^/h-PEO blends, indexed to the characteristic reflections of the Laves C14 and C15 phases; derivation of structural parameters for the core–shell–corona model; schematic illustration of the evolution of the core–shell–corona micelle structure with increasing *f*_*h‑PEO*_ (PDF)
