# Supplementary material for: Emergence of Frank–Kasper Phases from Chemically Simple Block Copolymer: Poly(ethylene oxide)-block-polyisoprene and Its Dry-Brush Blends
Source: Macromolecules. 2026 Feb 2;59(3):1529–41. doi: 10.1021/acs.macromol.5c03133 (PMC12895517; doi:10.1021/acs.macromol.5c03133)
Supplement: Supplementary file 1 [file ma5c03133_si_001.pdf]

Supporting Information

Emergence of Frank-Kasper Phases from Chemically Simple Block Copolymer: Poly(ethylene oxide)-*block*-Polyisoprene and Its Dry-Brush Blends

Zi-En Huang[1†], Yung-Chuan Chuang[2†], Yung-Chen Lin[1], Yu-Chuan Sung[2], Kai-Wei Luo[1], Jing-Cherng Tsai*[2], and Hsin-Lung Chen*[1]

[1] Department of Chemical Engineering, National Tsing Hua University, Hsinchu 300044, Taiwan

[2]Department of Chemical Engineering, National Chung Cheng University, Chiayi 621301, Taiwan

† These authors contributed equally to this work.

***Corresponding Author**

H.-L. Chen: hlchen@che.nthu.edu.tw

J.-C. Tsai: chmjct@ccu.edu.tw

1. Synthesis and characterizations of PEO-*b*-PI

The PEO-*b*-PI diblock copolymers were synthesized through a two-step process. In the first step, the PI block was prepared by living anionic polymerization of isoprene initiated by *tert*-butyllithium (*tert*-BuLi). In the second step, the living PI chains were coupled with tosyl-terminated PEO to produce the PEO-*b*-PI diblock copolymers. All reactions and manipulations were conducted in a standard Schlenk line or a dry box under a nitrogen atmosphere. Common reagents and solvents were purchased and used either as received or purified by distillation with calcium hydride or sodium/benzophenone. Isoprene, purchased from Sigma-Aldrich (purity >99.5%), was distilled from calcium hydride. *Tert*-butyllithium (1.9 M in heptane) was obtained from Sigma-Aldrich and used as received. Hydroxyl end-capped poly(ethylene oxide) (PEO) (M_n=1300g/mol, $Đ$=1.05) was purchased from Polymer Source, Inc.

Preparation of tosyl end-capped PEO. PEO-OH (5.0 g, 3.8 mmol) was dissolved in dry THF (30 mL) under a nitrogen atmosphere. Sodium hydride (0.37 g, 15 mmol, 4 equiv.) was then added slowly with continuous stirring. After complete dissolution, a solution of tosyl chloride (7.3 g, 38 mmol, 10 equiv.) in THF was added dropwise to the reaction mixture. The reaction was heated to 50 °C and stirred overnight.

Then, the reaction solution was quenched with excess hexane, resulting in the formation of a white polymer gel at the bottom of the flask. The gel was washed several times with 100 mL of hexane and then dried under vacuum. The final product, tosyl end-capped PEO, was obtained as a colorless polymer gel (5.5g, 97.2% yield). Figure S1 shows the respective ^{1}H NMR spectra (with inset showing the expanded region and the chemical assignments) of the produced tosyl end-capped PEO (M_n=1300 g/mol, $Đ$ =1.05).

Preparation of PEO-*b*-PI diblock copolymer. The synthesis of $PEO^{1.3}$-*b*-$PI^{3.15}$ is described here as a representative example to illustrate the general synthetic and characterization procedures for the diblock copolymers. The preparation was carried out in two steps. In the first step, living anionic polymerization of isoprene was initiated with *tert*-butyllithium (*tert*-BuLi) in benzene at room temperature to yield polyisoprene (PI) with a controlled molecular weight (M_n = 3150 g mol^{-1}, $Đ$ = 1.04). In the second step, tosyl-terminated PEO (0.5 g, M_n = 1300 g mol^{-1}, $Đ$ = 1.05) was dissolved in 10 mL of benzene and reacted with an excess of the living PI anion (1.7 mmol, 5.0 equiv.). An excess of the PI living chain was employed to ensure efficient substitution due to the gel-like physical state of the tosyl-terminated PEO, which otherwise hindered effective grafting. The reaction mixture was maintained at 50 °C for 8 days to ensure complete coupling.

After quenching with excess methanol, a light yellow polymer gel was observed at the bottom of the flask. It was then further washed twice with 20 mL of methanol. The resulting polymer gel containing PI and PEO-*b*-PI was subjected to column chromatography using a 1:1 (v/v) mixture of ethyl acetate (EA) and hexane as the eluent. During column chromatography, polyisoprene containing eluate passed through the column first, the PEO-*b*-PI can be selectively collected after the PI eluate and before the PEO eluate. The collected PEO-*b*-PI eluate was dried under vacuum overnight to provide a pure PEO-*b*-PI sample. Figure S2 compares the GPC elusion curves of the anionic PI (M_n = 3150 g/mol, $Đ$ = 1.04) with the PEO-*b*-PI (M_n = 4450 g/mol, $Đ$ = 1.05). Figure S3 shows the respective ^{1}H NMR spectra (with inset showing the expanded region and the chemical assignments) of the produced PEO-*b*-PI (M_n=4450 g/mol; $Đ$ =1.05).

Polymer analysis. The molecular weight and molecular weight distribution (MWD) of the synthesized polymers were determined by using gel permeation chromatography (GPC, Waters 1515-2414, USA) with a refractive index (RI) detector and a set of U-Styragel HT columns of 10^6, 10^5, 10^4, and 10^3 pore sizes in series. The measurements were taken at 35 °C using THF as a solvent. Polystyrene (PS) samples with narrow MWDs were used as the standards for calibration. The standards had an absolute molecular weight ranging from 980 to 2110000 g/mol; the *R* square of the ideal calibrated line was limited to up to 0.999.

All ^{1}H and ^{13}C NMR spectra were recorded on a Bruker AV-500 NMR spectrometer. The PEO-*b*-PI polymer samples were dissolved in $CDCl_3$; the spectra were recorded at a temperature of 50 °C.

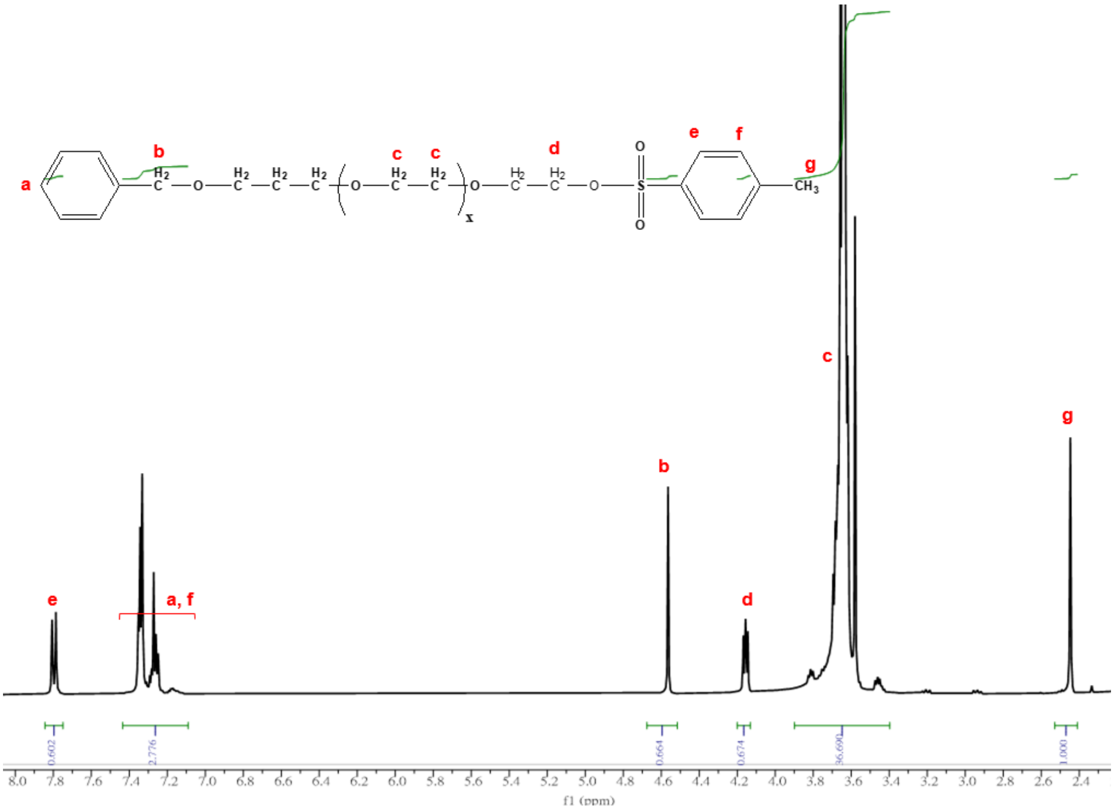

Figure S1. ^{1}H NMR (500MHz) of tosyl end-capped PEO, M_n=1300 g/mol, $Đ$ =1.05 (solvent, $CDCl_3$; temperature, 50 °C)

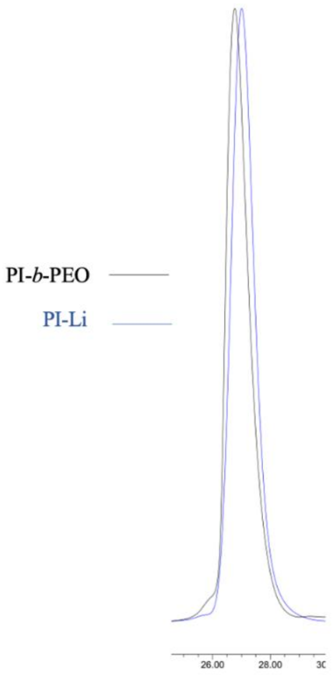

Figure S2. GPC curve comparison between anionic PI (M_n=3150 g/mol, $Đ$ =1.04) and PEO-*b*-PI (M_n=4450g/mol, $Đ$ =1.05) (solvent: THF; 35 °C).

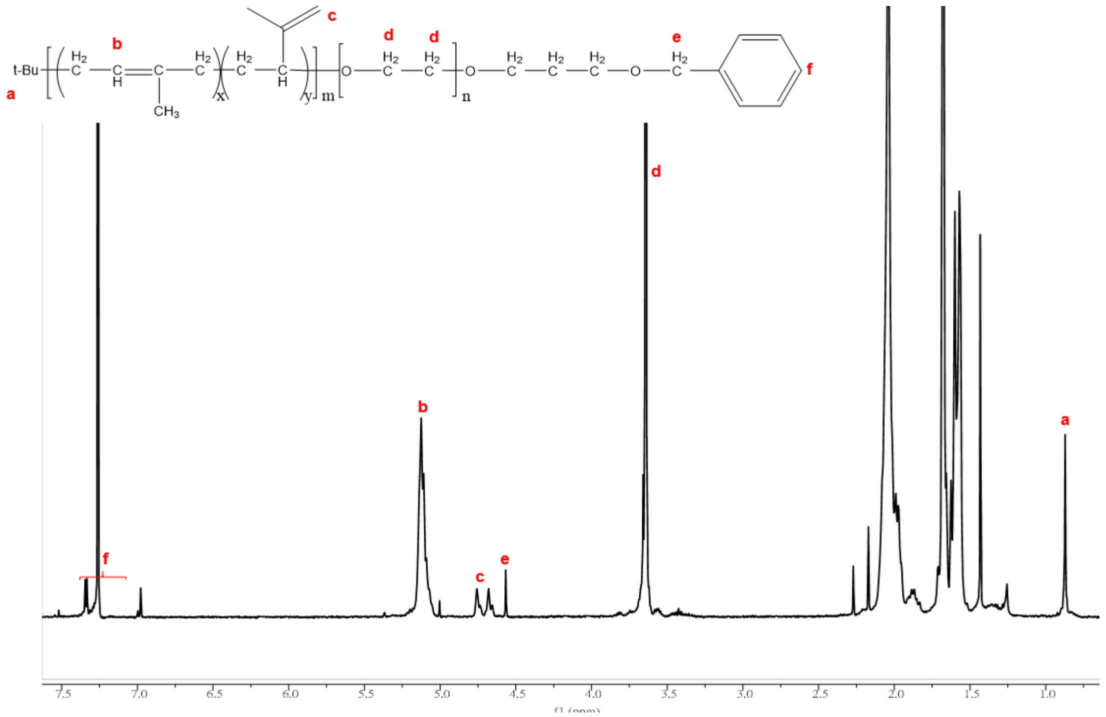

Figure S3. ^{1}H NMR (500MHz) of PEO-*b*-PI, M_n=4450 g/mol, $Đ$ =1.05 (solvent, $CDCl_3$; temperature, 50°C)

2. Wide-angle X-ray scattering (WAXS) profiles of neat PEO-b-PI and PEO-*b*-PI/h-PEO dry-brush blends equilibrated at 30 °C.

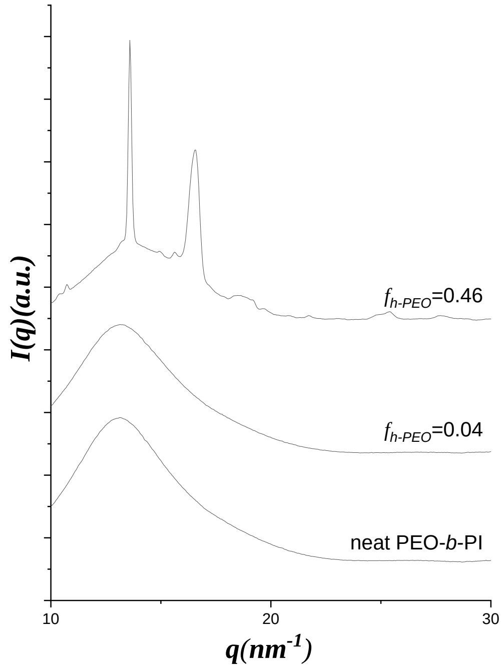

Figure S4. The WAXS profile of $PEO^{1.3}$-*b*-$PI^{4.5}$ and its blends with the overall h-PEO volume fraction $f_{h\text{-}PEO}$ = 0.04 and 0.46 collected at 30 °C. The samples had been equilibrated at 30 °C for three days. Except for blends with high h-PEO content ($f_{h\text{-}PEO}$ = 0.46), where macrophase separation occurred, the other two samples exhibited only a broad amorphous halo, indicating that the PEO components (both block PEO and h-PEO) confined within the micellar cores remained amorphous at room temperature. The suppressed crystallizability of PEO originated from strong spatial confinement within discrete microdomains, where crystallization required homogeneous nucleation. As demonstrated previously, the temperature required to initiate homogeneous nucleation of PEO confined in spherical domains can be as low as −30 °C, well below the temperatures explored in this study. Consequently, the ordered structures observed by SAXS were not perturbed by PEO crystallization.

3. Differential scanning calorimetry (DSC) evidence for dry-brush segregation in PEO-*b*-PI/h-PEO blends

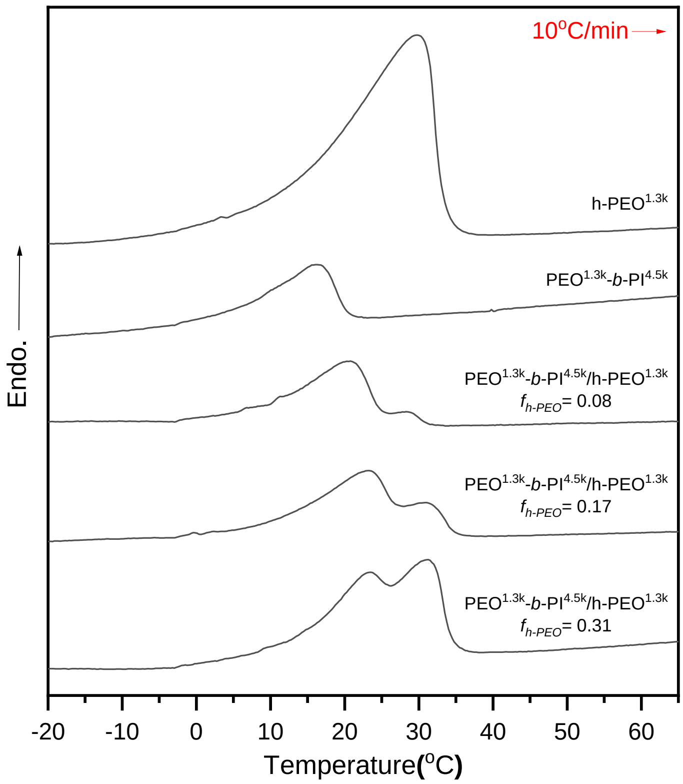

Figure S5. DSC heating thermograms obtained at a heating rate of 10 °C min^{-1} for neat h-PEO^{1}, neat PEO$^{1.3}$-*b*-PI$^{4.5}$ and PEO$^{1.3}$-*b*-PI$^{4.5}$/h-PEO blends at various h-PEO volume fractions ($f_{h\text{-}PEO}$). The melting temperature (T_m) of neat h-PEO is approximately 30 °C, whereas that of the PEO block (b-PEO) in neat PEO-*b*-PI is around 15 °C. The blends crystallized at -30 °C exhibit two distinct melting endotherms: a lower-temperature peak associated with the melting of the b-PEO and a higher-temperature peak corresponding to the melting of h-PEO. The absence of a single, merged melting transition indicates that the b-PEO and h-PEO do not co-crystallize or mix homogeneously at the molecular level. Instead, h-PEO remains spatially segregated within the PEO microdomain, consistent with dry-brush behavior and supporting a three-layer micellar architecture comprising an h-PEO core, a b-PEO-enriched shell, and a PI corona.

4. SAXS peaks of the PEO$^{1.3}$-*b*-PI$^{4.5}$/h-PEO blend with $f_{h\text{-}PEO}$ = 0.03, indexed to the characteristic reflections of the Frank-Kasper σ phase

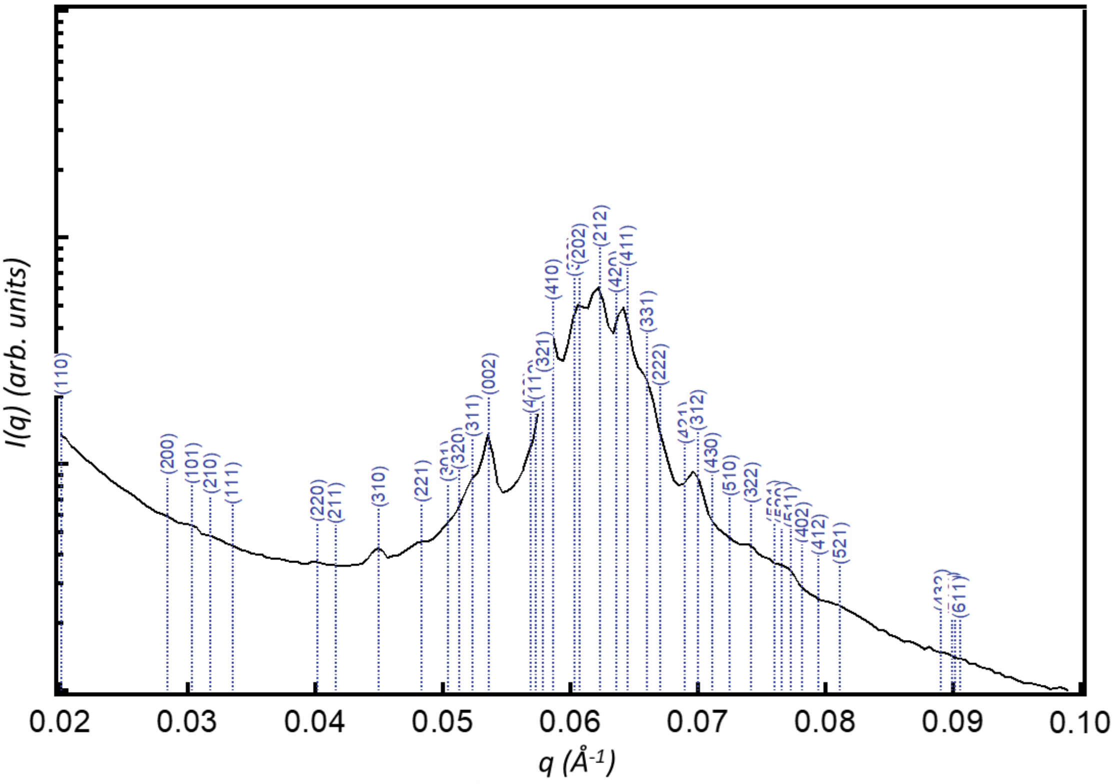

Figure S6. Peak indexing of the SAXS profile for the PEO$^{1.3}$-*b*-PI$^{4.5}$/h-PEO blend with the overall h-PEO volume fraction $f_{h\text{-}PEO}$ = 0.03, collected at 50 °C after annealing for 60 min. The diffraction peaks can be indexed to the characteristic reflections of the Frank–Kasper σ phase, corresponding to a tetragonal unit cell with lattice parameters *a*=44.25 nm and *c*=23.42 nm.

5. SAXS peaks of the $PEO^{1.3}$-*b*-$PI^{4.5}$/h-PEO blends, indexed to the characteristic reflections of the Laves C14 and C15 phases

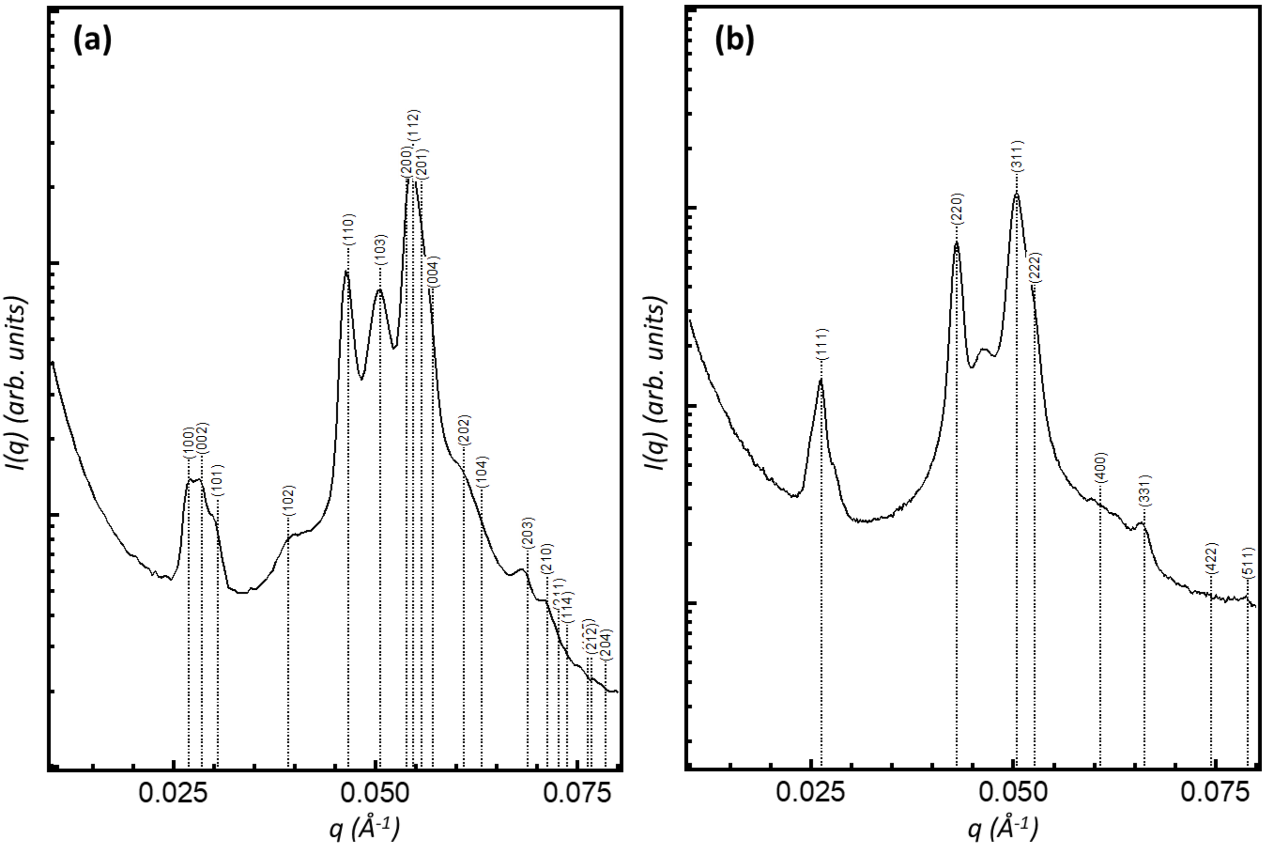

Figure S7. Peak indexing of the SAXS profiles collected at 30 °C for the $PEO^{1.3}$-*b*-$PI^{4.5}$ /h-PEO blend with the overall h-PEO volume fractions: (a) $f_{h\text{-}PEO} = 0.04$, corresponding to the Laves C14 phase($P6_3/mmc$) with the hexagonal lattice parameters a = 26.95 nm and c = 44.23 nm; and (b) $f_{h\text{-}PEO} = 0.17$, corresponding to the Laves C15 phase ($Fd\bar{3}m$) with the cubic lattice parameter a = 39.94 nm.

6. Derivation of structural parameters for the core-shell-corona model

To analyze the distribution of h-PEO, b-PEO, and PI components within the micelles, we adopted a core-shell-corona geometric model in which each micelle is represented by an equivalent sphere. This construction is used solely for geometric bookkeeping and visualization and does not constitute a thermodynamic model.

6.1 Average Micelle Volume

The average micelle volume, $\langle V_m \rangle$, was determined from the unit-cell volume of the corresponding lattice identified by SAXS and the number of micelles per unit cell:

$$\langle V_m \rangle = \frac{V_{\text{cell}}}{n}$$

where V_{cell} is the crystallographic unit-cell volume and n is the number of micelles per unit cell. The equivalent micelle radius R_m is then given by:

$$R_m = \left(\frac{3\langle V_m \rangle}{4\pi} \right)^{1/3}$$

6.2 Partitioning of Micelle Volume

The average micelle volume was partitioned into three components:

$$\langle V_m \rangle = \langle V_{\text{c,h-PEO}} \rangle + \langle V_{\text{s,b-PEO}} \rangle + \langle V_{\text{cn,PI}} \rangle$$

where $\langle V_{c,h\text{-}PEO} \rangle$ is the volume of the h-PEO core, $\langle V_{s,b\text{-}PEO} \rangle$ is the volume of the b-PEO shell, and $\langle V_{cn,PI} \rangle$ is the volume of the PI corona. Each contribution was calculated from the known volume fraction, as follows:

$$<V_{cn,PI}> = < V_m > (1 - f_c)$$

$$< V_{s,b-PEO} > = < V_m > f_{b-PEO}$$

$$< V_{c,h-PEO} > = < V_m > f_{h-PEO}$$

where f_c is the overall volume fraction of the core composing of b-PEO and h-PEO.

6.3 Characteristic Length Scales

The radius of the h-PEO core $R_{c,h\text{-}PEO}$ was obtained from:

$$R_{\mathrm{c,h\text{-}PEO}} = \left(\frac{3\langle V_{\mathrm{c,h\text{-}PEO}} \rangle}{4\pi} \right)^{1/3}$$

The thickness of the b-PEO shell $l_{s,b\text{-}PEO}$ was calculated as:

$$< I_{s,b-PEO} >= (\frac{3 < V_m > f_c}{4\pi})^{\frac{1}{3}} -< R_{c,h-PEO} >$$

The thickness of the PI corona $l_{cn,PI}$ was calculated as:

$$< I_{cn,PI} >= \left(\frac{3 < V_m >}{4\pi}\right)^{\frac{1}{3}} -< R_{c,h-PEO} > -< I_{s,b-PEO} >$$

7. Schematic illustration of the evolution of the core-shell-corona micelle structure with increasing $f_{h\text{-}PEO}$

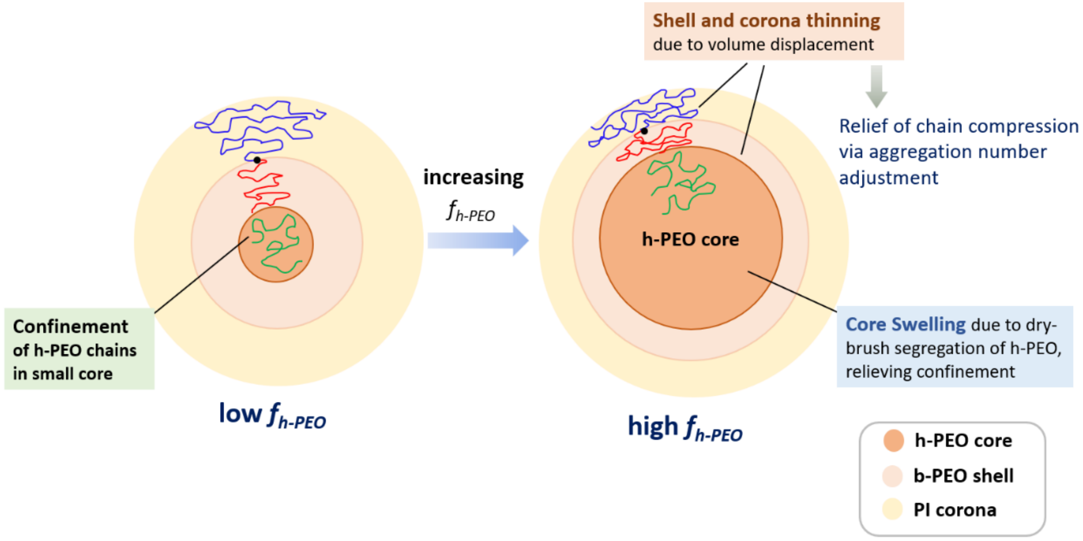

Figure S8. Schematic illustration of the evolution of the core–shell–corona micelle structure with increasing $f_{h\text{-}PEO}$. Increasing h-PEO content leads to swelling of the h-PEO core driven by dry-brush segregation, accompanied by progressive thinning of the b-PEO shell and PI corona due to volume redistribution and chain stretching. The schematic highlights the three competing geometric effects: core swelling, shell thinning, and corona thinning, together with aggregation-number adjustment, which underlie the systematic and nonmonotonic structural trends shown in Figure 7. This illustration is intended as a qualitative guide.
